# Near-Infrared Absorption Features of Triplet-Pair States Assigned by Photoinduced-Absorption-Detected Magnetic Resonance

**DOI:** 10.1021/acs.jpclett.2c03665

**Published:** 2023-02-27

**Authors:** Ryan D. Dill, Gajadhar Joshi, Karl J. Thorley, John E. Anthony, Brian Fluegel, Justin C. Johnson, Obadiah G. Reid

**Affiliations:** † University of Colorado Boulder, Department of Chemistry, Boulder, Colorado 80309, United States; ‡ National Renewable Energy Laboratory, Golden, Colorado 80401, United States; ¶ University of Kentucky Center for Applied Energy Research, Lexington, Kentucky 40511, United States; § Department of Chemistry, University of Kentucky, Lexington, Kentucky 40506, United States; ∥ Renewable and Sustainable Energy Institute, University of Colorado, Boulder, Colorado 80309, United States

## Abstract

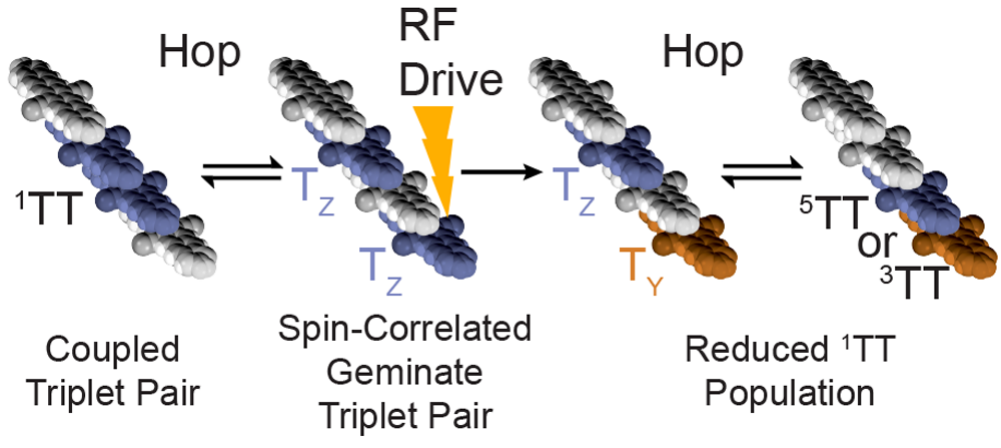

Singlet fission proceeds through a manifold of triplet-pair states that are exceedingly difficult to distinguish spectroscopically. Here, we introduce a new implementation of photoinduced-absorption-detected magnetic resonance (PADMR) and use it to understand the excited-state absorption spectrum of a tri-2-pentylsilylethynyl pentadithiophene (TSPS-PDT) film. These experiments allow us to directly correlate magnetic transitions driven by RF with electronic transitions in the visible and near-infrared spectrum with high sensitivity. We find that the new near-infrared excited-state transitions that arise in thin films of TSPS-PDT are correlated with the magnetic transitions of T_1_, not ^5^TT. Thus, we assign these features to the excited-state absorption of ^1^TT, which is depleted when T_1_ states are driven to a spin configuration that forbids subsequent fusion. These results clarify the disputed origin of triplet-associated near-infrared absorption features in singlet-fission materials and demonstrate an incisive general purpose tool for studying the evolution of high-spin excited states.

Singlet fission (SF) is a multiexciton generation process wherein a singlet excited state on one chromophore, S_1_, partitions its energy with a nearby chromophore in its ground state, S_0_, to generate two triplet excited states, T_1_ + T_1_. As a downconversion process, SF can be used in photovoltaics to reduce thermalization losses,^1−3^ but recent work has also suggested that intermediate triplet-pair states, particularly ^5^TT, may have quantum information (QI) applications,^4−8^ since these may form with high initial spin polarization. Distinct pair states, ^*n*^TT, form when two adjacent triplet states experience a strong interchromophore exchange interaction, such that pairs with different spin multiplicities, *n* = 1, 3, or 5, possess different energies. Although SF initially proceeds through spin-allowed interconversion to ^1^TT, the net-triplet and net-quintet states, ^3^TT and ^5^TT, often play an important role in the dynamics but are exceedingly difficult to distinguish due to their electronic similarity.

In principle, the intertriplet interactions make the triplet-pair states distinguishable from T_1_,^9^ but in practice, it is difficult to accurately assign optical absorption features. Here, we introduce a new implementation of photoinduced-absorption-detected magnetic resonance (PADMR) spectroscopy and use it to directly correlate photoinduced absorption spectra with spin transitions to identify the optical contributions of triplet-pair states in a polycrystalline thin film of tri-2-pentylsilylethynyl pentadithiophene (TSPS-PDT; Figure 1).^10,11^ PADMR is a type of population-detected magnetic resonance, or magnetic action spectrum—magnetic-sublevel transitions drive changes in an observed electronic-state concentration by modifying one or more electronic-state interconversion rate constant. In the case of PADMR, we measure the change in photoinduced (or excited state) optical absorption of a sample caused by RF- or microwave-induced transitions within the magnetic state manifold. This detection mechanism affords a much more versatile tool than the more commonly employed photoluminescence-detected magnetic resonance (PLDMR), as it can be applied to any system with high-spin excited states, not only those kinetically coupled to luminescent states—a point of particular importance for studying singlet-fission systems, which do not usually emit. Although PADMR experiments have been described before,^12−30^ our PADMR spectrometer (diagrammed in Figures 1 and S2) differs markedly. It operates up to very high modulation frequencies (200 MHz) and permits measurements of the PADMR signal as a function of RF-drive frequency (*f*-PADMR), allowing access to short time scales and low (including 0) magnetic fields, in addition to the more common scan variables, probe wavelength (λ-PADMR) and magnetic field (*B*-PADMR).^17,19^ Especially for disordered samples, *f*-PADMR at zero magnetic field offers strong signals, good spectral resolution, and extremely simple interpretation. In addition, advances in available computer equipment in the 10–20 years since this technique was regularly used have allowed us to implement powerful 2D scan modes for correlating different scan variables that have not been previously demonstrated. See the Supporting Information section S1 for full experimental details. Applying this powerful tool to thin films of TSPS-PDT reveals strong magnetic resonance (MR) transitions associated with free triplet, T_1_, and weak MR transitions associated with small populations in ^5^TT. Importantly, the photoinduced absorption features in the near-infrared (NIR), which do not appear in solution and have been commonly assigned to triplet-pair states,^9,10,31,32^ are correlated with transitions in T_1_. This surprising observation is most likely explained by kinetic coupling between T_1_ and the magnetically dark ^1^TT state via recombination of spin-correlated geminate triplet pairs—leading us to assign the NIR PA features observed here to ^1^TT.

**Figure 1:**
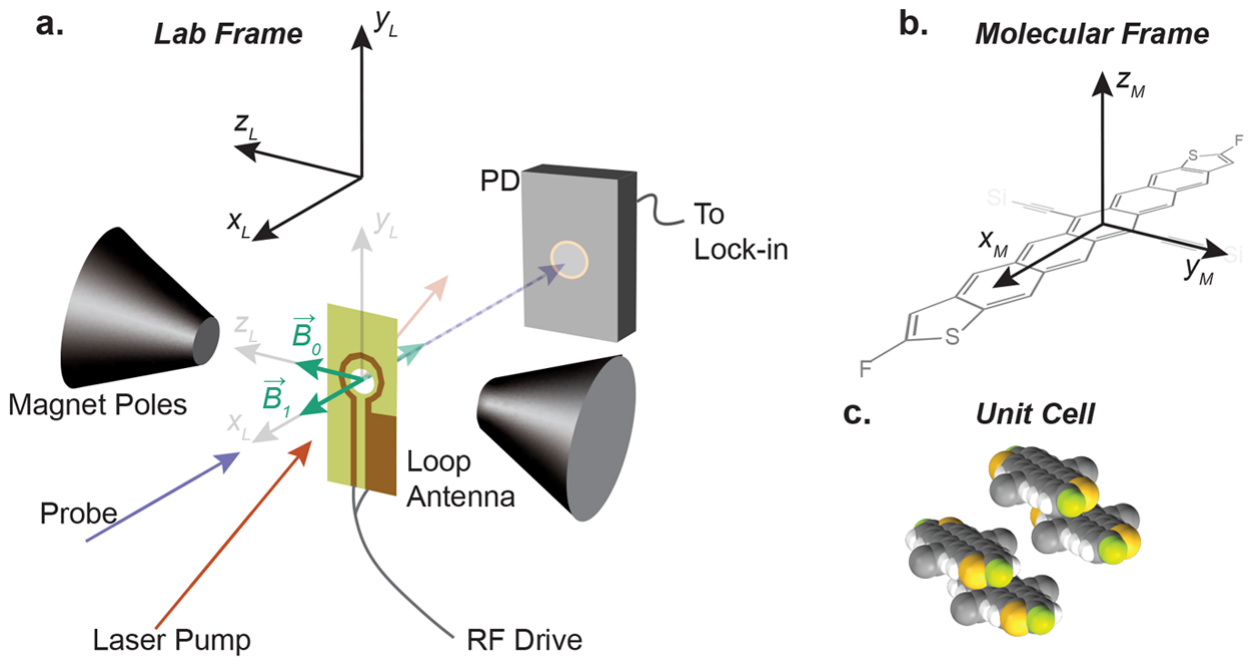
The PADMR spectrometer. (a) Lab-frame view of the PADMR spectrometer. The sample is mounted to a PCB loop antenna, inside an optical cryostat, between the pole tips of an electromagnet. The probe beam passes through the center of the RF antenna, and its intensity is measured by a photodetector. A laser is overlapped with the probe. For PA experiments, only the laser is modulated. For PADMR experiments, only the RF is modulated. See SI section S1.2 for details. (b) The molecular reference frame commonly used for acenes is assumed valid here.^33^ (c) The unit-cell for crystallites of TSPS-PDT, first reported in ref (10), is reproduced here. The reduced symmetry of TSPS-PDT, relative to standard acenes, means that there are four unique nearest-neighbor-pair conformations, two of which are shown. To aid in visualization, the trialkylsilyl substituents are not included.

We begin by discussing the photoinduced absorption (PA) spectroscopy of TSPS-PDT films, as it is essential background for understanding the PADMR results. PA spectroscopy probes the system’s *electronic* dynamics under modulated visible light excitation, or pump, and is the frequency-domain analog to pulsed-beam transient absorption spectroscopy, more typically carried out in the time-domain. For details on the experimental setup and interpretation, see the Supporting Information section S1.

*PA features of TSPS-PDT*. Figure 2a shows the absorption spectrum of a TSPS-PDT film at room temperature. Like the one in ref (10), it is red-shifted and broadened relative to the solution-phase absorption due to strong interchromophore interactions. Figure 2b shows the PA spectrum for the same sample, the lock-in signal associated with an amplitude-modulated laser pump recorded as a function of probe wavelength (temperature ≈ 5–10 K, 642 nm excitation, 1 kHz laser-modulation frequency, *f*_*mod*_^*opt*^). It is similar to the transient absorption spectra in ref (10), showing a ground-state bleach (GSB) near 500 nm, strong PA features with partially resolved vibronic progression from 550 to 650 nm, and PA in the NIR from 900 to 1200 nm. The additional GSB features are more difficult to discern but appear as weak dips in the spectrum around 725 and 840 nm. Reduced ground-state absorption around 750 nm appears as a small hump in the PA spectrum. The slight mismatch between steady-state absorption features and their corresponding PA features likely comes from the different temperatures used and site selectivity in the PA data due to laser photoexcitation. The vibronic progression peaking at 600 nm is typical of triplet-containing states in PDT films but does not easily distinguish T_1_ from the various triplet-pair states. The NIR PA features were previously attributed to triplet-pair states, based largely on their absence in solution-phase triplet spectra;^10^ their assignment is discussed further below.

**Figure 2:**
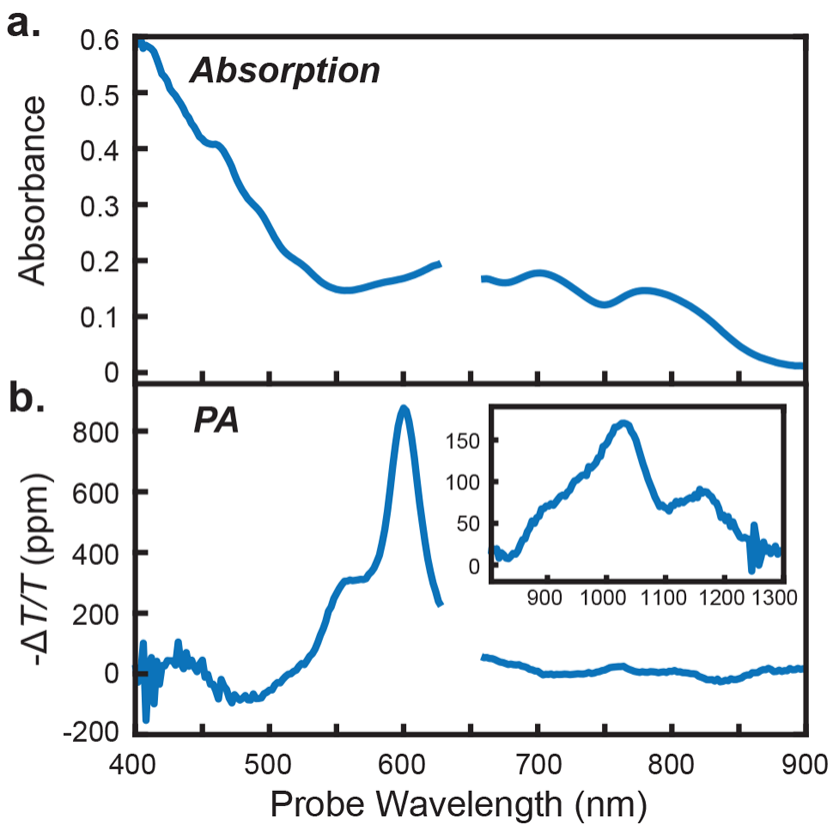
Steady-state and photoinduced absorption spectra for a TSPS-PDT film. (a) Absorption spectrum (−log(*T*/*T*_0_)) measured at room temperature with the experimental setup described in the text. A constant value (0.25) was subtracted to correct for scattering. The gap in the spectrum is where the notch filter absorbs. (b) The PA spectrum (642 nm pump wavelength, pump intensity ≈ 2 W/cm^2^, *f*_*mod*_^*opt*^ = 1 kHz, temperature ≈ 5–10 K) shows strong absorption peaking at 595 nm, associated with triplet states. The modulation frequency, *f*_*mod*_^*opt*^ = 1 kHz, is low, so the spectrum approximately represents steady-state PA. This spectrum has been rephased by ϕ = +22.9° to put most of the signal into a single channel (the rephasing process is discussed in section S1.2.5). Inset: PA signals in the NIR are also clear. Experimental parameters for both data sets are the same, except that an 800 nm long-pass filter was added for the NIR data. This spectrum has been rephased by ϕ = −70°.

Under conditions of low pump-modulation frequency, *f*_*mod*_^*opt*^, such as in Figure 2b, the signal is dominated by species with long lifetimes. At higher *f*_*mod*_^*opt*^, those signals are diminished—measuring the PA spectrum as a function of *f*_*mod*_^*opt*^ informs on electronic dynamics (Figures S10 and S11). Reproducing the entire modulation frequency dependence for TSPS-PDT requires at least five decay time constants. The longest three lifetimes (τ_1,2,3_ = 94, 42, and 3.5 μs) are all associated with very similar optical spectra (see SI Figures S11 and S12). The strong similarity in the PA spectra associated with these three time constants (SI Figure S12) suggests they are all associated with triplet-*containing* states (i.e., T_1_ or ^*n*^TT). The remaining signal at high *f*_*mod*_^*opt*^ is described by two time constants, τ_4,5_ = 430 and 1.8 ns; these time constants are associated with very different spectral shapes, dominated by broad indistinct PIA overlapping with sharp GSB features. The 1.8 ns time constant is probably related to the instrument bandwidth limit and therefore serves as an upper bound on the corresponding state lifetime. The observed lifetimes of all states depend on pump intensity and applied magnetic field (see SI Figures S13–S15). They are also much longer than those reported in ref (10), most likely due to the much lower temperatures used here (∼10 vs 300 K). Specific assignment of the two short-lived spectra (430 and 1.8 ns decay times) is not immediately clear and will require further investigation, as they do not resemble anything from our prior room-temperature TA studies.^10^ We thus focus the remainder of the discussion on the long-lived species observed at low pump-modulation frequencies.

As in prior transient absorption studies, the PA dynamics and spectra alone cannot be used to assign the features we observe to specific magnetic species. We turn to PADMR spectroscopy to directly correlate electronic transitions with the magnetic transitions to which they are kinetically coupled, in particular the NIR PIA features from 900 to 1200 nm.

*Free triplet signatures in *f*-PADMR*. The *f*-PADMR spectrum of TSPS-PDT probed at the typical triplet PA feature (600 nm) at zero magnetic field (RF-modulation frequency, *f*_*mod*_^*RF*^ = 2.99 kHz) is shown in Figure 3a. The three strongest features at 85, 990, and 1077 MHz are associated with transitions between sublevels of a triplet state, which we characterize in terms of its zero-field splitting parameters, *D* and *E*.^35^ A fit gives |*D*| = 1033 MHz and |*E*| = 41.6 MHz (Figure S16).

**Figure 3:**
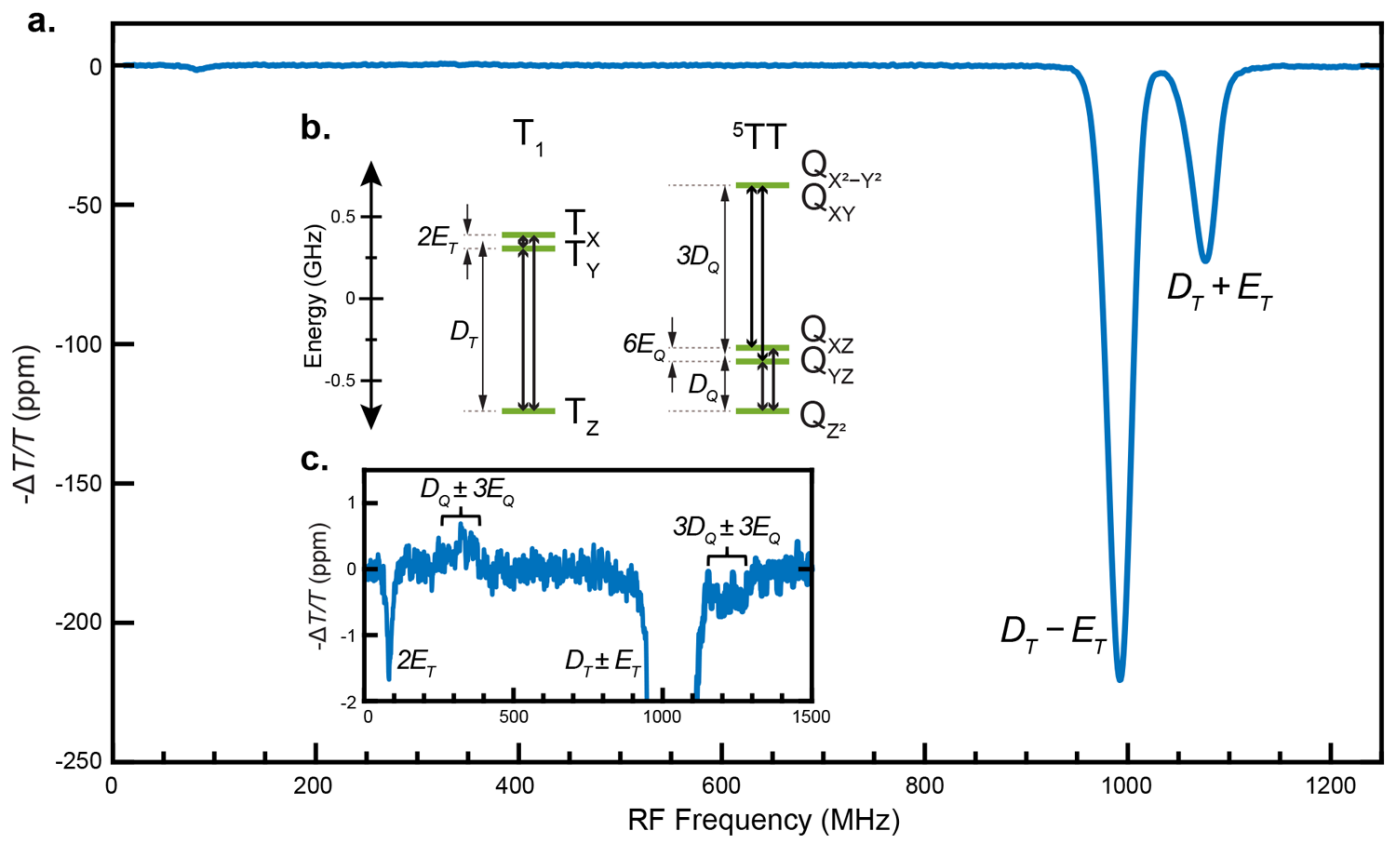
The PADMR spectrum of TSPS-PDT is dominated by triplet signatures. (a) Detecting at the triplet PA’s peak, the *f*-PADMR spectrum of a TSPS-PDT film shows three main peaks (85, 990, and 1077 MHz), corresponding to transitions within the triplet manifold, T_1_ (600 nm probe, 642 nm continuous pump, pump intensity ≈ 15 W/cm^2^, 2.99 kHz RF-modulation frequency, zero magnetic field, temperature ≈ 5–10 K). The negative signals indicate that PA intensity is reduced by RF drive. These data have been rephased by ϕ = +29.8° to maximize the amplitude in a single channel (the rephasing process is discussed in SI sections S1.2.5 and S1.2.6). (b) Sublevel energy diagram for T_1_ (left) and ^5^TT (right) in the zero-field basis. Arrows show observed transitions. The T_1_ splittings are from a fit, with |*D*_*T*_| = 1033 MHz and |*E*_*T*_| = 41.6 MHz. The ^5^TT sublevels are named as in ref (4), and the splittings shown disregard anisotropic exchange.^34^ (c) Much weaker features are assigned to quintet transitions.

The simplest assignment of these magnetic transitions is to T_1_, corresponding to 2*E*_*T*_, *D*_*T*_ – *E*_*T*_, and *D*_*T*_ + *E*_*T*_. While ^3^TT and ^5^TT are expected to show transitions at similar frequencies (Figure 3b) major contributions from these species can be ruled out. It is unlikely that the observed peaks are primarily composed of ^5^TT transitions because ^5^TT typically displays comparably strong MR features at intermediate frequencies, corresponding to Q_Z^2^_ ↔ Q_YZ_ and Q_Z^2^_ ↔ Q_XZ_. Transitions at such frequencies *are* observed (Figure 3c; see below) but are orders of magnitude weaker. Assignment of the strong features to ^3^TT is similarly unlikely, though it cannot be distinguished on the basis of the spectra. ^3^TT is expected to decay via the spin-allowed “triplet-channel annihilation” (TCA) pathway, ^3^TT → T_1_ + S_0_^36−39^ and is unlikely to form with high yield to begin with due to symmetry considerations.^4,34,36,40^

The PADMR signals in Figure 3a are negative, −*ΔT*/*T* < 0, indicating that driving these MR transitions *reduces* photoinduced absorption, corresponding to a reduced concentration of the probed state(s). In general, this is caused by sublevel-dependent dynamics, such as a faster decay rate in the product sublevel. The specific origins of the negative PADMR signal are discussed below.

Further interpretation of peak amplitudes in Figure 3 is not straightforward for several reasons. Even the *relative* peak amplitudes are complicated functions of all the dynamical processes with magnetic-sublevel-dependent electronic-state interconversion rates—they are not simply proportional to sublevel population differences; they may be distorted by instrument sensitivity that varies with RF-drive frequency, *f*_*Drive*_^*RF*^ (see SI section 2.3.4). They may also depend on crystallite orientation bias. The last point is an important nuance: although the transition *frequencies* of a triplet state do not depend on molecular orientation at zero magnetic field, their relative intensities do. Each transition is polarized along a different molecular axis;^41,42^ their intensities depend on their projection onto the driving RF magnetic field, *B⃗*_1_.

*Weak ^5^TT transitions in the *f*-PADMR spectrum*. Closer inspection of the *f*-PADMR spectrum (Figure 3c) reveals weak positive features at 250–400 MHz and negative ones at 1150–1250 MHz; we assign these to ^5^TT. The 250–400 MHz features correspond to Q_Z^2^_ ↔ Q_YZ_ and Q_Z^2^_ ↔ Q_XZ_ (Figure 3b). Those transitions are characteristic of ^5^TT, since they are centered at *D*_*Q*_ ≈ *D*_*T*_/3, where *D*_*Q*_ is the zero-field splitting parameter for ^5^TT.^34,43,44^

We assign the 1150–1250 MHz features to the four transitions that couple Q_YZ_ and Q_XZ_ to Q_X^2^-Y^2^_ and Q_XY_. This assignment is implied by their similar amplitudes compared to the 250–400 MHz features and supported by the slight shift relative to the T_1_ features. The relationships between the ^5^TT and T_1_ zero-field splitting parameters, *D*_*Q*_ = *D*_*T*_/3 and *E*_*Q*_ = *E*_*T*_/3, are only exact when the isotropic exchange, *J*_*TT*_, is the only interaction between the two triplets. In real systems, sublevel energies are also affected by anisotropic interactions; for triplet-pairs, the most important is the magnetic dipole–dipole interaction, analogous to the zero-field splitting but between the triplets in the pair.^8,34,43−45^ Although we have assigned the weak features to groups of quintet transitions, their low signal-to-noise ratio precludes a more specific and quantitative assignment.

It is clear from these experiments that both T_1_ and ^5^TT are populated by photoexcitation, as detected using the 600 nm PA feature commonly associated with the net-triplet population. In what follows, we turn to the NIR PA feature at 1000 nm, seeking to clarify its assignment and take advantage of its electronic-state specificity.

*NIR PA correlates with T_1_ MR*. In Figure 4, we show PADMR data for TSPS-PDT for the region surrounding the T_Z_ ↔ T_Y_ transition. Figure 4a shows the signal magnitude as a function of both *f*_*Drive*_^*RF*^ and probe wavelength. Vertical slices in the pseudocolor plot correspond to *f*-PADMR spectra at fixed probe wavelengths; horizontal slices are λ-PADMR spectra at fixed RF-drive frequencies. Figure 4b shows λ-PADMR spectra for selected RF-drive frequencies, extracted from a global fit reconstruction of the phase-optimized PADMR signal (, where *X*′ is the real part of the complex-valued signal after rephasing; see SI section S1.2.5 for a discussion of the rephasing process). Figure 4c shows the λ-PADMR basis spectra used to model the full data; each is associated with a Gaussian line shape along the *f*-PADMR dimension centered at the labeled frequency.

**Figure 4:**
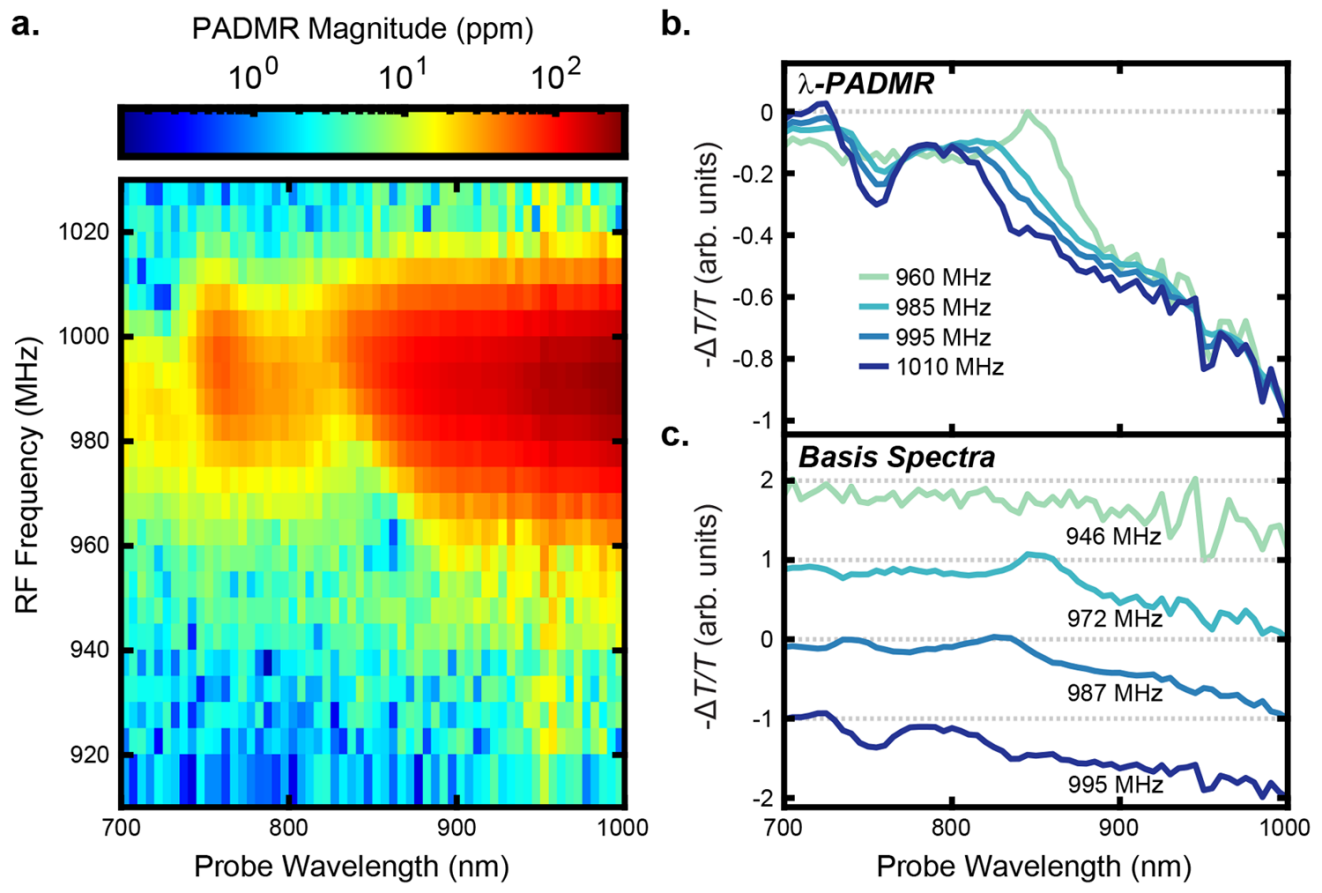
*f* × λ-PADMR correlates near-IR PA with T_1_ MR. (a) The PADMR signal magnitude, , shows strong signals when probing the 1000 nm PA (Figure 2) and driving the T_Z_ ↔ T_Y_ transition (642 nm pump wavelength, pump intensity ≈ 15 W/cm^2^, *f*_*mod*_^*RF*^ = 2.99 kHz, temperature ≈ 5–10 K). Slight asymmetry shows that the PA and MR spectral positions are correlated. (b) λ-PADMR spectra demonstrate shifting PA intensity as a function of *f*_*Drive*_^*RF*^. They are slices taken from a global fit of the phase-optimized PADMR data (applied phase, ϕ = +34.2°; see SI section S1.2.5 for a discussion of the rephasing process). (c) The λ-PADMR basis spectra obtained by the fit. All are most intense near 1000 nm. A superimposed positive feature is seen at 850 nm when driven by 972 MHz RF and likely corresponds to a GSB (RF increases ground-state population). It shifts to bluer wavelengths with increasing *f*_*Drive*_^*RF*^. A similar shift is seen in a GSB near 740 nm, and pronounced shifts are seen when probing near the 600 nm PA (SI section S2.3.1).

All of the λ-PADMR spectra in Figure 4b,c show the previously discussed NIR PA (Figure 2b, inset), as their signal continues increasing beyond 1000 nm. The PADMR signal is strongest near *f*_*Drive*_^*RF*^ = 990 MHz, the T_Z_ ↔ T_Y_ transition, indicating that that the 1000 nm PA belongs to T_1_*or a state kinetically linked to it*. Its negative sign means that driving the transition *reduces* the probed state population. These observations combined with a consideration of past literature strongly suggest we assign the NIR PA feature in TSPS-PDT to ^1^TT. Recall that PADMR signals arise through the influence that magnetic transitions have on the concentration of the probed electronic state: the state you drive with RF is not necessarily identical to that you detect electronically—they need only be kinetically coupled in a sublevel-dependent manner. While assignment to T_1_ remains possible, ^1^TT is more likely in light of prior work. It was previously assigned to a triplet-pair state,^10^ and similar features in other acene derivatives have been assigned explicitly to ^1^TT based in part on their similarity to other singlet transitions and their absence in solution-phase triplet spectra, wherein isolated chromophores cannot possess energetically accessible ^1^TT.^9,31,32^ This assignment is discussed more in the Supporting Information. Importantly, it is consistent with a geminate triplet-pair recombination model, which we discuss in the following section.

*Spin-correlated geminate triplet pairs*. Figure 5a shows an overall kinetic scheme consistent with singlet fission in TSPS-PDT, which has antiferromagnetic TT exchange coupling,^10^ including both triplet-pair separation and loss pathways. In general, dissociation of a triplet-pair state produces a geminate pair of triplets, ^1^TT → T_1_ + T_1_. Although electronically decoupled, geminate triplet pairs may remain spin-correlated long after dissociation, especially at low temperatures where spin–lattice relaxation is slow, as illustrated for ^1^TT dissociation in Figure 5b,c. These have been previously described for singlet-fission systems, including in one of our own works.^46−48^

**Figure 5:**
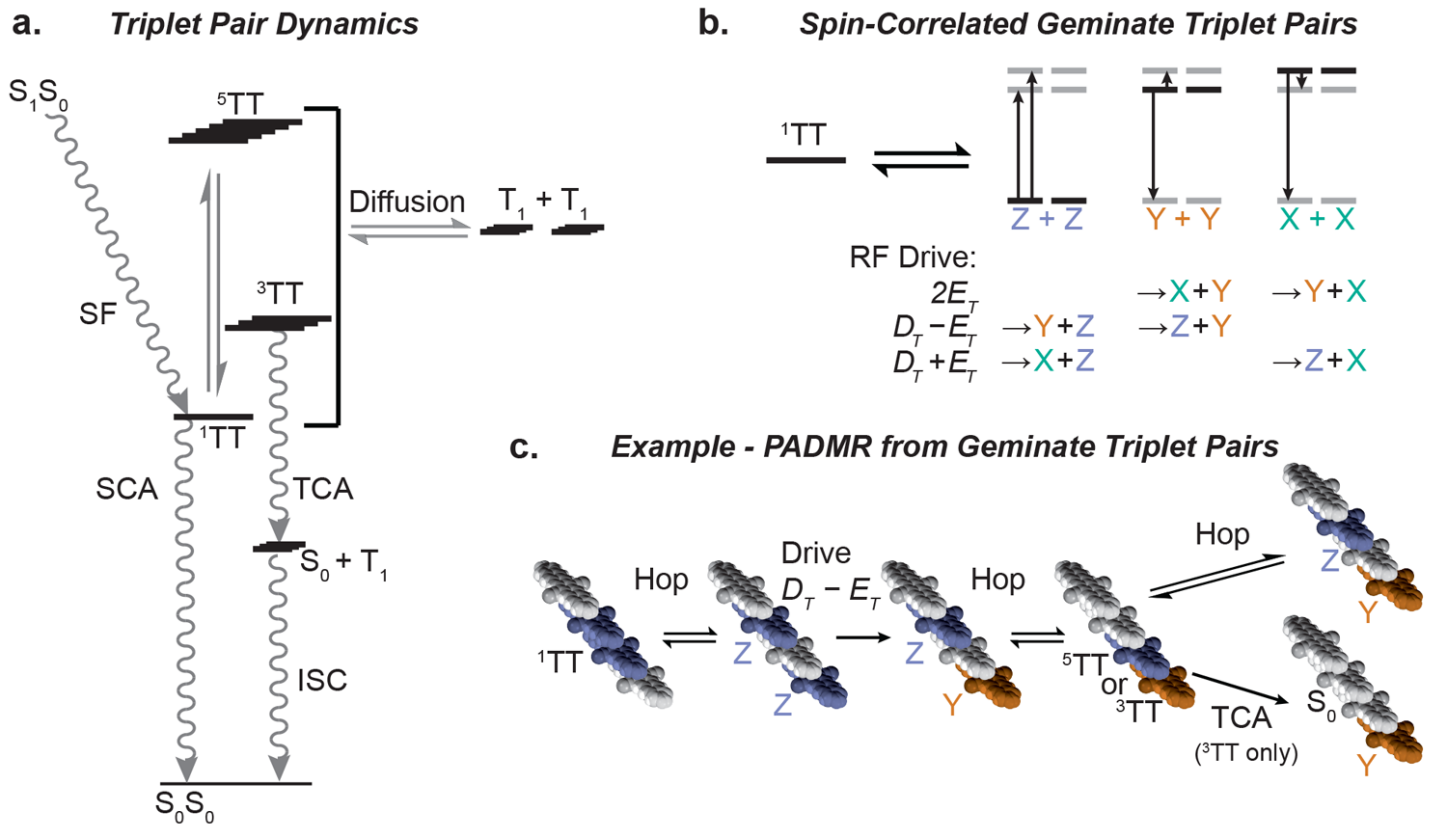
Triplet-pair model for low-temperature TSPS-PDT films. (a) Dynamical model showing triplet-pair formation (SF), diffusion, and deactivation via singlet-channel and triplet-channel annihilation (SCA and TCA), T_1_ deactivation through intersystem crossing (ISC), and ^1^TT ⇌ ^5^TT equilibration (energy gaps are not to scale). Due to a large antiferromagnetic *J*_*TT*_,^10^^5^TT forms in low yields. (b) ^1^TT dissociation yields either T_X_ + T_X_, T_Y_ + T_Y_, or T_Z_ + T_Z_. X, Y, and Z correspond to T_X_, T_Y_, and T_Z_. RF absorption drives one partner into a different sublevel, preventing reassociation to ^1^TT. (c) An example pathway in which geminate triplet pairs produce a PADMR signal. Molecular stacks are extended along the crystal’s *a*-axis. ^1^TT formation is followed by a hop. Spatial separation electronically decouples the triplets (*J*_*TT*_ → 0), but they remain spin-correlated: here, as T_Z_ + T_Z_. Decoupled triplets can absorb RF photons: here, RF with frequency *D*_*T*_ – *E*_*T*_ drives a T_Z_ → T_Y_ transition. With reassociation, strong electronic coupling returns; the new pair configuration has net-triplet or net-quintet character. Both ^3^TT and ^5^TT may dissociate again, but ^3^TT can also deactivate via TCA.

The spin correlation within geminate triplet pairs arises from the original coupled-pair (^*n*^TT) wave function, which can be represented by linear combinations of zero-field states in the uncoupled pair basis.^34,36,49^ If the triplets are on parallel copies of the same chromophore, then ^1^TT = 1/√3(|T_X_T_X_⟩ + |T_Y_T_Y_⟩ + |T_Z_T_Z_⟩).^34,36,49^ Thus, each ^1^TT dissociation event should yield two of the same sublevel (Figure 5b), assuming the superposition state decoheres rapidly. In the ensemble, dissociation should yield equal populations in T_X_, T_Y_, and T_Z_ (unequal populations may form through other means, including spin–lattice relaxation). A population with equal sublevel occupations cannot overall absorb or emit RF, precluding observation of an EPR signal. However, individual spins *can*. If one triplet from a geminate triplet pair such as T_Z_ + T_Z_ absorbs an RF photon (Figure 5b,c), it transitions to a different sublevel, yielding a mismatch such as T_Z_ + T_Y_ that has no overlap with the ^1^TT wave function. Reassociation in this case will not form ^1^TT or the ^5^TT sublevels that couple most strongly to it, Q_Z^2^_ and Q_X^2^-Y^2^_.^4,34^ The result is a steady-state reduction in ^1^TT population upon RF drive, just as observed in Figure 4.

Unlike the ^1^TT-specific PA at 1000 nm, we expect ^1^TT and T_1_ to show overlapping PA at 600 nm. But just like the NIR-probe PADMR, those PADMR signals are negative as well (Figures 3a and S20). This suggests that the net effect of RF drive is to push population into spin configurations that favor ^3^TT—for example, ^3^TT_X_ = 1/√2(|T_Y_T_Z_⟩ – |T_Z_T_Y_⟩)—thus promoting overall triplet population relaxation via TCA (see Figure 5a,c).^36^

Thus, our data is consistent with a triplet population dominated by long-lived-spin-correlated geminate triplet pairs that only rarely interact nongeminately. Systems dominated by nongeminate triplet-pair association give the opposite trend, where RF drive increases the rate of triplet fusion.^46,48^ This is because nongeminate triplet pair encounters with singlet character may fuse, depleting those configurations from the population at steady state. The result in such a case is that RF drive will push the population toward more singlet pair configurations on average.

It is clear that geminate triplet-pair recombination is dominant in our data, since the 1000 nm probe PADMR signal attributed to ^1^TT is negative, −*ΔT*/*T* < 0. This is not surprising: the low-temperature, low-dimensional transport and large antiferromagnetic *J*_*TT*_ would naturally discourage dissociation, slow down diffusion, and encourage reassociation so that the uncoupled T_1_ live transiently and rarely diffuse far enough to encounter a nongeminate partner. However, some nongeminate triplet recombination probably does occur, as implied by the shortened PA lifetimes with increased laser intensity (600 nm probe, Figure S13). Since ^1^TT is probably a trap (lowest energy excited state), it may be surprising that ^1^TT → T_1_ + T_1_ dissociation would occur at all. But ^1^TT forms with a large excess of energy (∼0.5–1 eV); the time it takes for this energy to dissipate is probably sufficient for some pairs to escape the trap. The excess energy may also be needed to produce the small but observable quintet populations (Figure 3c).

*Correlations between PA and MR frequencies*. With the data in Figure 4b, we demonstrated a kinetic link between T_1_ MR and a PA most likely belonging to ^1^TT. The *f* × λ-PADMR data also shows that the MR transition frequencies are correlated with the optical absorption wavelengths (Figure 4). Increasing *f*_*Drive*_^*RF*^ between 960 and 1010 MHz coincides with blue-shifting features in the λ-PADMR spectra. Perhaps most obvious is the sharp superimposed peak that starts at 850 nm (960 MHz) and blue-shifts by as much as 50 nm (90 meV). A similar feature (of opposite sign) is seen in the PA spectrum (Figure 2b) and is likely a GSB. Given that assignment, the positive sign of the superimposed GSB means that the ground-state population is increased by RF drive. The spectral shift is large, but entirely within the expected distribution, given the breadth of the film absorption spectrum (Figure 2a). The implication is that the inhomogeneous broadening in the MR lines and the optical absorptions is related: there is a positive correlation between the optical and magnetic resonance frequencies. Similar behavior is seen when probing the 600 nm PA (Figure S20): high RF-drive frequencies are associated with bluer PA.

Linear correlations between MR and optical line positions have been previously discussed for other triplet states^50−56^ and derive from inhomogeneities that perturb the electronic environment. The inhomogeneities must also be relatively static: rapidly sampling different environments produces line-narrowing like is seen with zero-field splittings in solution-phase EPR and NMR spectra. Although we cannot yet identify the specific inhomogeneity responsible, its presence in our data demonstrates an important aspect of PADMR—it is powerfully selective.

In conclusion, we have implemented a new variant of photoinduced-absorption-detected magnetic resonance spectroscopy and used it to study the correlations between magnetic and transient optical spectra in a unique singlet-fission material, polycrystalline TSPS-PDT. We identify strong magnetic resonance transitions associated with uncoupled triplet states, T_1_, which are accompanied by weak signatures of coupled ^5^TT states. Furthermore, we find that the near-infrared photoinduced absorption features previously assigned to a variety of triplet-pair states are correlated with magnetic transitions in the T_1_ manifold. These observations are consistently explained by a model where the free triplet population is dominated by spin-correlated geminate triplet pairs, and driving magnetic transitions promotes ^3^TT configurations that have a rapid relaxation pathway while simultaneously forbidding fusion back to ^1^TT. We thus assign the near-infrared optical absorption to ^1^TT in this system.

These results show the long-persistent spin correlations present in this and similar singlet-fission materials at low temperature and demonstrate a unique and versatile way to detect them, even absent net population level spin polarization. While persistent spin correlation is generally useful for quantum information applications, it can be problematic in other applications like triplet state harvesting for photovoltaics, since geminate triplet-pair recombination facilitates deactivation. On the other hand, loss of spin correlation should be accelerated at higher temperatures, possibly mitigating this issue. Outside the context of singlet fission, these studies have laid foundations for detailed understanding of essentially any spin-active excited state through correlations between optical absorption and magnetic resonance.

## Supporting Information Available

Detailed exposition of sample preparation, detailed discussion of all spectroscopic methods, instrument details, data analysis methods, RF coupling loop simulations, supplemental exploration of laser power and magnetic field dependence of the signals, and various supplemental discussions of points discussed more briefly in the main text (PDF)
