# Supplementary material for: Near-Infrared Absorption Features of Triplet-Pair States Assigned by Photoinduced-Absorption-Detected Magnetic Resonance
Source: J Phys Chem Lett. 2023 Feb 27;14(9):2387–94. doi: 10.1021/acs.jpclett.2c03665 (PMC10009807; doi:10.1021/acs.jpclett.2c03665)
Supplement: Supplementary file 1 — jz2c03665_si_001.pdf [file jz2c03665_si_001.pdf]

Supporting Information for:

Near-Infrared Absorption Features of Triplet Pair States Assigned by Photoinduced Absorption-Detected Magnetic Resonance

Ryan D. Dill,[†] Gajadhar Joshi,[‡] Karl J. Thorley,[¶] John E. Anthony,[¶,§] Brian Fluegel,[‡] Justin C. Johnson,[‡,‖] and Obadiah G. Reid[*,‖,‡]

†*University of Colorado Boulder, Department of Chemistry, Boulder, CO, 80309, United States*

‡*National Renewable Energy Laboratory, Golden, Colorado 80401, United States*

¶*University of Kentucky Center for Applied Energy Research, Lexington, Kentucky 40511, USA*

§*Department of Chemistry, University of Kentucky, Lexington, Kentucky 40506, USA*

‖*Renewable and Sustainable Energy Institute, University of Colorado, Boulder, Colorado 80309, USA*

E-mail: obadiah.reid@colorado.edu

Contents

S1 Experimental Methods **S3**

S1.1 Sample Preparation and Notes S3

S1.2 Spectroscopic Methods S3

S1.2.1 PA Spectroscopy S4

S1.2.2 PADMR Spectroscopy S7

S1.2.3 Instrument Details S11

S1.2.4 Experimental Details S17

S1.2.5 Rephasing Lock-in Signals S18

S1.2.6 Data Analysis and Presentation Details S19

S2 Supplementary Discussions **S25**

S2.1 PA Spectroscopy of TSPS-PDT S25

S2.2 f-PADMR Spectroscopy of TSPS-PDT S30

S2.2.1 The PADMR signal's frequency-dependent phase S31

S2.3 $f \times \lambda$-PADMR for TSPS-PDT S34

S2.3.1 Probing the 600 nm PA S36

S2.3.2 Correlations derive from inhomogeneity S39

S2.3.3 Optical/MR correlations are normal. S39

S2.3.4 RF simulations of the broadband RF coupling loop. S44

References **S46**

S1 Experimental Methods

S1.1 Sample Preparation and Notes

Three TSPS-PDT films were simultaneously prepared with a blade-coater in a glovebox. Blade-coating was done at 50°C, using 15μL of a TSPS-PDT in chlorobenzene solution (40 mg/mL), with a blade speed of 32 mm/s at blade height of 200 μm. Dry films were stored in the dark under ambient air. Similar TSPS-PDT samples have been previously shown to be polycrystalline and partially ordered.[1] The samples showed no visible signs of discoloration after several days of operation, but the scattering gradually increased, reducing the detected probe intensity. That likely suggests optically induced annealing.

As this is a neat organic material with low dielectric constant, the steady-state population of photogenerated charge carriers is expected to be far lower than that of triplets. Our data does not show evidence for charge carriers or polarons, and while that does not rule out their existence, the role of triplet-polaron quenching is likely to be negligible, unlike systems with intentional charge injection or charge separation.[2,3]

S1.2 Spectroscopic Methods

The methods used here are detailed in Ref. 4. We include an abridged version of those discussions here. All data presented in this article were collected with a PADMR spectrometer we constructed, which uses a lock-in amplifier to record the change in transmitted light, ΔT, associated with modulated laser excitation or RF drive. The same spectrometer, modified for photoluminescence detection, was also used in Ref. 5.

Many different experiments can be performed with the PADMR spectrometer, but they can be broadly categorized into two groups. In photoinduced absorption (PA) experiments, the lock-in is referenced to the pump laser modulation frequency, f_{mod}^{opt}; ΔT_{PA} is the change in transmitted light induced by photoexcitation.[6,7] In photoinduced absorption detected

magnetic resonance (PADMR) experiments, on the other hand, the lock-in is referenced to the RF modulation frequency; ΔT_{MR} is the change in transmitted light due to the presence of the RF drive. The type of signal (PA or PADMR) is defined by which input is modulated. For either type, when the input is modulated at low frequency, the signal approaches its steady state limit. Dynamical information can be obtained by recording the signal as a function of the modulation frequency.

An excellent discussion of modulation-frequency-resolved photoluminescence experiments is presented by Lakowicz;[8] although the experiments discussed there are somewhat different, understanding those simpler experiments serves as important background. For readers seeking to construct an ODMR or PADMR, we also recommend References 9–11.

S1.2.1 PA Spectroscopy

A schematic diagram of our spectrometer set up for PA spectroscopy is shown in Fig. S1. We define PA experiments as those which (1) detect transmitted (or absorbed) light, (2) use an amplitude-modulated laser pump, and (3) lock-in at the laser modulation frequency. The probe beam intensity is continually converted to an electrical signal by a photodetector. A high intensity, modulated, pump laser generates excited state populations. Although it is far less common, PA spectroscopy is the frequency-domain analogue of transient absorption spectroscopy (TA). One of the most important advantages of PA spectroscopy is that it is amenable to lock-in amplifier detection, facilitating high-sensitivity measurements.[6,7] Quasi-steady-state (qSS) PA spectra are obtained by modulating the laser pump at a low frequency (the low frequency limit, $f_{mod} = 0$, defines steady-state), and recording the PA signal as a function of probe wavelength.

Although absorption is the property of interest, the change in transmitted light, ΔT, is typically what is actually measured. However, ΔT measurements often carry a lot of unwanted information about the instrument (for example, the shape of the lamp spectrum is not usually of interest). That can largely be eliminated by recording ΔT as a relative

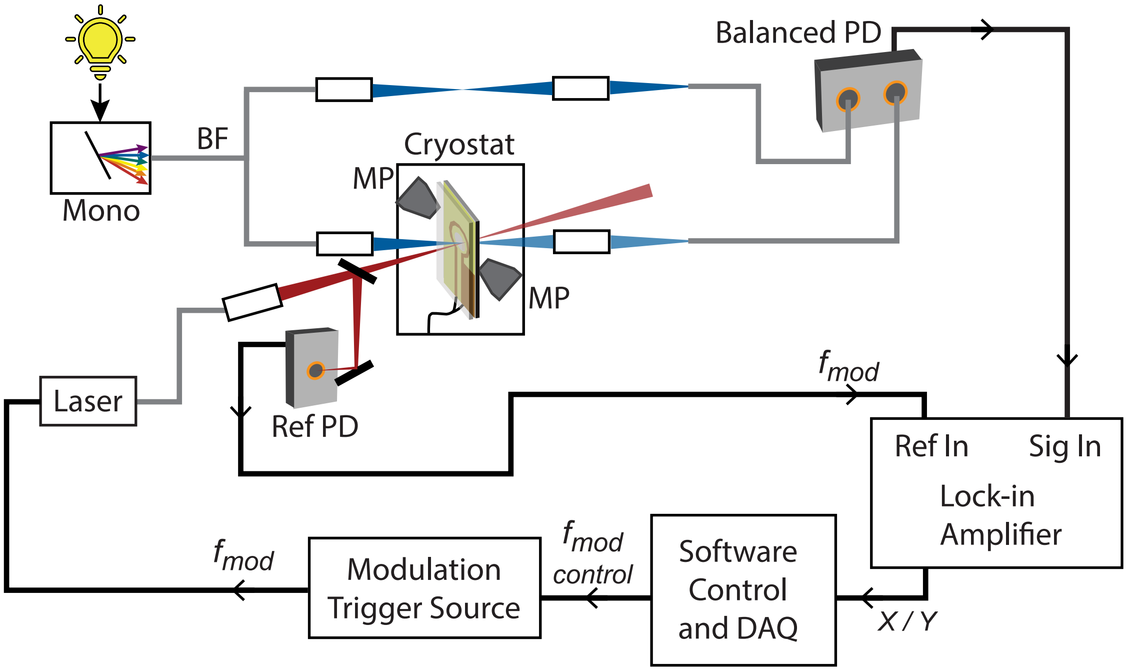

Figure S1: A schematic diagram of our spectrometer being used for PA spectroscopy. Optical fibers are in gray, coaxial and data connections are in black. The optical probe is wavelength-selected by a monochromator. The monochromator output is focused onto a bifurcated optical fiber (BF) to produce a signal and a reference path. The signal path is focused through the sample, which is mounted inside a cryostat. Signal and reference are refocused onto optical fibers, and detected by a balanced photodetector. Balancing is not usually necessary for PA experiments. The pump laser is digitally modulated; triggering is provided by a separate source. The modulated laser is passed through a 10% beam-sampler, and the transmitted beam is overlapped with the probe in the sample. The reflected beam is directed to a reference photodiode, which is used as a reference input for the lock-in amplifier. The reference photodiode is needed to match the signal/reference time delays, when the modulation-frequency-dependence is of interest. The lock-in amplifier demodulates the oscillating part of the photodiode signal. PA spectra are collected by measuring the lock-in signal as a function of wavelength; PA lifetimes are estimated by measuring the lock-in signal as a function of laser modulation frequency. Some components are excluded from the diagram for simplicity.

measurement — divide it by T. The final version of the PA signal then is $-\frac{\Delta T}{T}$, where the negative sign is not necessary but makes comparison to true absorption measurements easier, since $\Delta A \approx \frac{1}{\ln 10}\left(\frac{-\Delta T}{T}\right)$. Although it is preferable to simultaneously measure T and ΔT, practical limitations sometimes require that they are measured separately (for example, splitting the signal can increase noise).

Background signals caused by scattering and spontaneous photoluminescence, among other things, can be eliminated with a dual-modulation scheme, wherein the probe is also modulated with an optical chopper.[4,6] This is most useful for qSS spectra, rather than lifetime measurements, because varying one of the modulation frequencies complicates detection and interpretation of dual-modulation signals.

For single-modulation experiments, when the modulation frequency is increased above the qSS limit ($f_{mod} \ll 1/\tau_{response}$, where $\tau_{response}$ is the signal's longest response time constant), the sample's non-instantaneous response becomes important. When the modulation period is comparable to the response time constant, the signal ΔT is demodulated and phase-shifted relative to the qSS signal.[8] PA data measured as a function of modulation frequency therefore provides dynamical information and can be used to measure lifetimes.[8,12–14] While demodulation and phase-shifts (R and Θ, respectively) may be slightly more intuitive, lock-in amplifiers typically measure the in-phase and out-of-phase contributions to the modulated signal, X and Y.

When measured as X and Y, the modulation-frequency-dependence effectively provides the real and imaginary parts of the Fourier transform of a TA experiment. An inverse Fourier transform would give the time-domain impulse response, but it usually makes more sense to analyze this type of data directly in the frequency domain. For single-exponential decay dynamics with time constant τ, $X(f_{mod})$ and $Y(f_{mod})$ look respectively like absorption and dispersion lineshapes centered at $f_{mod} = 0$ ($\omega = 2\pi f_{mod}$):[8]

$$
\begin{aligned}
X(\omega) &= a\left(\frac{\tau}{1+\omega^2\tau^2}\right) \\
Y(\omega) &= a\left(\frac{\omega\tau^2}{1+\omega^2\tau^2}\right)
\end{aligned} \tag{S1}
$$

These equations require, of course, that signal and reference path lengths are well-matched. Otherwise, the time-delay will lead to a background phase offset that varies with modulation frequency.

S1.2.2 PADMR Spectroscopy

PADMR measures RF- or microwave-induced changes to the light transmitted through a photoexcited sample. When the probe wavelength is tuned to an absorption feature, PADMR effectively measures the change in electronic state concentration caused by driving a high-spin state between magnetic sublevels.

Figure S2 shows one of the simplest experimental setups for measuring PADMR signals (a diagram of the sample space in our PADMR spectrometer is also shown in Figure 1a of the main text). The optical layout is the same as for a PA experiment (Fig. S1), but for PADMR experiments the laser is not modulated; the laser produces a steady-state excited-state population. An amplitude modulated RF source is added to drive magnetic sublevel transitions, and the lock-in detects at the RF modulation frequency, f^{RF}_{mod}. A magnet is added to control sublevel splittings, and is an important feature of any general-purpose ODMR or PADMR spectrometer. A cryostat capable of cooling the sample to near-liquid-helium temperatures is also typically necessary, since thermal energy encourages spin-lattice relaxation, which attenuates ODMR signals.[9,15]

To understand how PADMR signals arise, consider the simple case in which the magnetic sublevels' optical spectra are indistinguishable. For example, for an intersystem crossing triplet T_1, only one PA band would be seen, consisting of the superimposed PA from all three sublevels, T_X, T_Y and T_Z. This is often a good assumption, but it is not essential for observation of an ODMR signal.[9] For simplicity, we also disregard spin-lattice relaxation

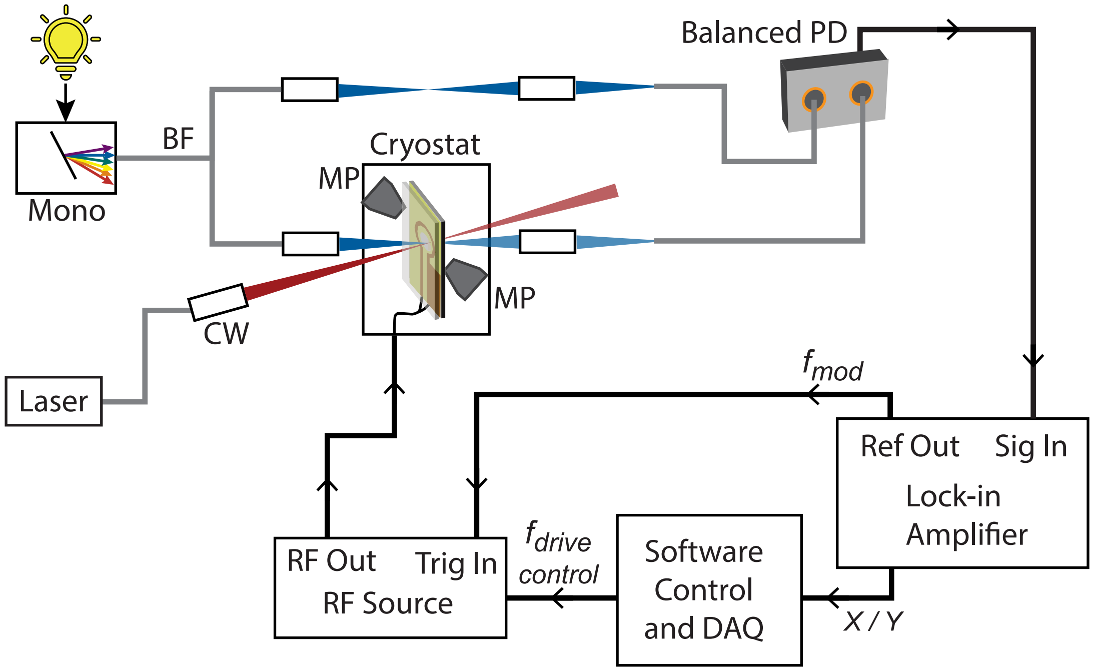

Figure S2: A schematic diagram of our spectrometer being used for PADMR spectroscopy in single-modulation mode. Optical fibers are in gray, coaxial and data connections are in black. The probe setup is the same as for PA (see Fig. S1). Balanced-detection is often necessary for PADMR experiments. The pump laser is run in CW mode; it is *not* modulated. The reference PD assembly is not relevant, so it is excluded from the diagram. The RF drive signal is provided by a broadband RF source, and connected to the cryostat's RF feedthroughs. It is modulated internally, but the modulation trigger is provided by the lockin's digital output. The modulated RF irradiation is provided by a home-etched PCB loop antenna or a resonator for high-field operation. The cryostat is equipped with an electro-magnet (magnet poles, MP, are shown). The magnetic field is also software controlled. The lock-in amplifier demodulates the oscillating part of the photodiode signal produced by the modulated RF drive. PADMR spectra are collected by measuring the lock-in signal as a function of RF drive frequency (f-PADMR), magnetic field (B-PADMR), or probe wavelength (λ-PADMR).

(SLR), which transfers population between the magnetic sublevels and tends to produce a Boltzmann distribution. Even at low temperatures, SLR rates can be non-negligible, but for this general discussion of how PADMR signals arise SLR can be neglected.

To observe a PADMR signal then requires that driving a magnetic resonance changes the overall electronic state population, $T_1 = T_X + T_Y + T_Z$. It is not obvious that this should actually happen — driving a magnetic resonance (MR) transfers population between two sublevels; it does not directly change the overall triplet population. However, the magnetic sublevels are each unique, which manifests in unique formation and relaxation rates, and consequently unequal populations. The overall triplet population does not change immediately in response to RF-drive. Driving a magnetic resonance perturbs the sublevel populations, and since their electronic formation and decay rates are different the overall triplet population is different when the RF-drive is present. A steady-state interpretation is helpful, and is valid when low modulation frequencies are used. In that limit, the presence of the laser produces steady-state populations in all states; the presence of both the laser *and* the RF-drive produces a distinct steady-state.

The steady-state picture just described is demonstrated in Figure S3. First, when the laser is turned on at $t = 0$, excited states start to populate (Fig. S3b), simultaneously reducing the ground state population. Those populations will eventually become time-independent (steady-state), and are determined by the system's natural relaxation dynamics. The ΔT described by this first steady-state approach is the PA signal (Fig. S3c); it does not contribute to the PADMR signal, since the actual PADMR experiment uses continuous laser pump. When the RF-drive is turned on, in this case resonant with the $T_Y \leftrightarrow T_Z$ transition, it drives population between sublevels (Fig. S3b). But those transitions are not measured directly — they can only be detected if they affect the electronic dynamics. For example, note that in Figure S3c, the triplet population approaches its final value slowly — the system takes time to reach a new steady state. The PADMR signal is the small difference between the steady-state PA signals with and without the RF drive.

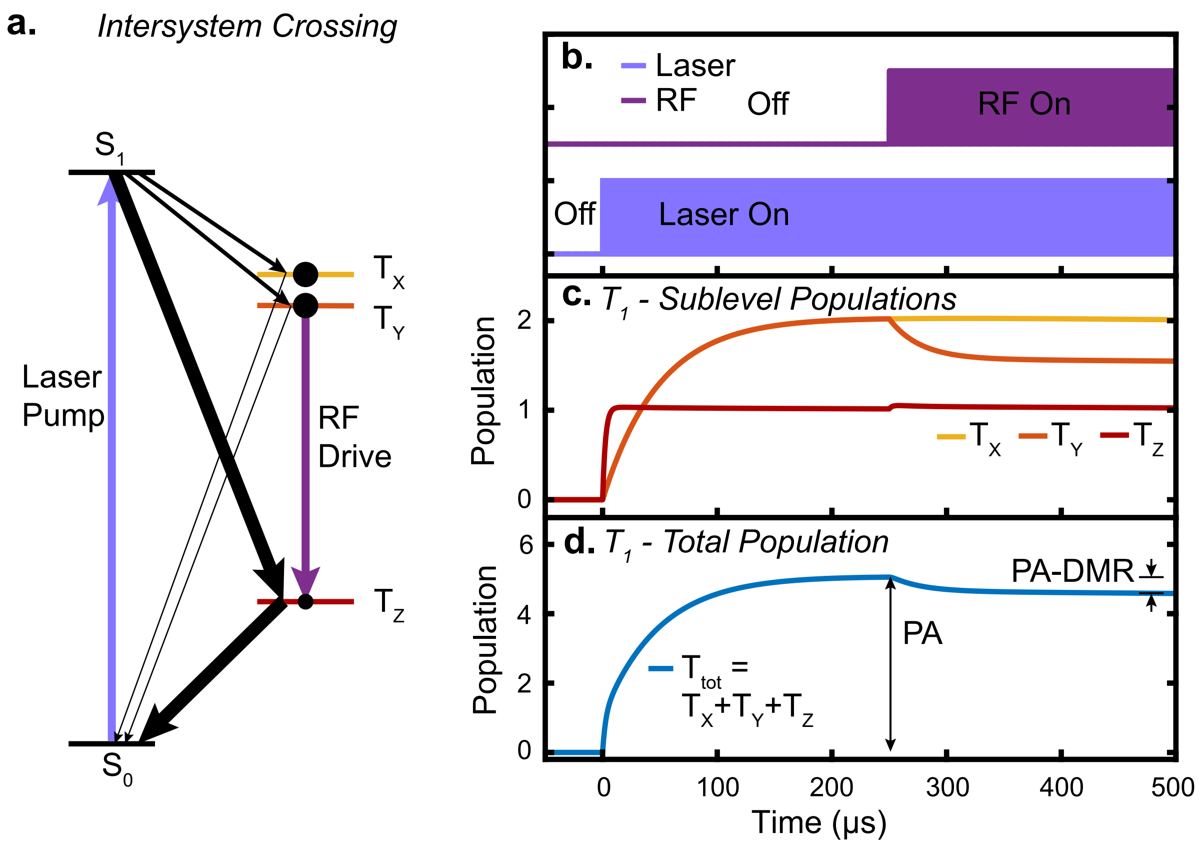

Figure S3: A simple picture of steady-state PADMR signals for an intersystem crossing triplet. The basic PADMR experiment uses a CW laser pump and modulated RF. **a.** Kinetic model for a hypothetical intersystem crossing triplet in which T_X and T_Y are kinetically identical, but T_Z is unique. Thicker arrows qualitatively represent faster rate constants. Although the formation rate for T_Z is faster than for T_X or T_Y (10 times faster), its decay is faster too (20 times faster). The sublevel-dependent rate constants lead to different populations, enabling resonant RF to drive population between them. Spin-lattice relaxation is omitted for simplicity. **b.** A hypothetical laser pump and RF-drive timing schedule. The laser turn-on is not part of the PADMR experiment. **c.** When the laser is continuously on, the triplet sublevels reach steady-state populations determined by their unique formation and decay rates. With its faster dynamics, T_Z more rapidly approaches steady-state. Since the *relative* decay rate for T_Z is faster than its relative formation rate, it builds up a smaller steady-state population than T_X and T_Y. Turning on the RF drive transfers population to the less-populated state, T_Z. **d.** The PADMR signal is the RF-induced change in PA, which relates to the *overall* triplet population. The overall triplet population is perturbed on the steady-state approach timescale, which is dictated by the various interconversion rates.

Finally, RF drive cannot perturb just one electronic state population. It must perturb two or more states, or none at all. For example, in Figure S3, the decrease in triplet population caused by the RF-drive must be compensated by increased populations in at least one other state. Since PADMR signals contain contributions from all electronic states whose populations are perturbed, probe-wavelength resolution (λ-PADMR) produces an optical difference spectrum, similar to transient absorption. Unlike transient absorption, however, the ground state absorption spectrum is not necessarily one of the superimposed spectra, although it often will be. This complexity is important to consider — a probe-wavelength resolved PADMR spectrum is not necessarily the PA spectrum of the magnetically driven state.

S1.2.3 Instrument Details

The cryostat (Montana Instruments Cryostation) is a closed-cycle helium cryostat. The sample is cooled conductively. We record temperatures as low as 5 K, but these are not measured at the sample surface, which may be slightly higher. We know that heating from the laser and the RF occur, but it is not clear how much they affect the average temperature within the probe spot. We expect that the sample temperature is < 10 K. Since the main reason that we use low temperatures is to enhance PADMR signals by reducing spin-lattice relaxation, the exact value is not critical, as long as it is “low enough”.

The electromagnet (Magneto-optic option for the Cryostation) can produce fields up to 450-500 mT. Custom pole tips with a larger tip-tip separation and a custom radiation shield were purchased from Montana instruments to free up space within the sample chamber.

The optical layout is pictured in Figure S4. The probe is produced by a lamp coupled to a monochromator. We have used both xenon arc lamps and tungsten-halogen lamps, but prefer tungsten-halogen lamps. Although arc lamps produce brighter output at visible and near UV wavelengths, their output is highly structured above ~ 800 nm. This makes it difficult to collect reliable wavelength-resolved data. Further, the lack of UV light in the output of

tungsten lamps prevents solarization damage to the optics.

To enable balanced detection, we removed the monochromator's exit slit, and instead image the output onto a bifurcated optical fiber. The bifurcated fibers are displaced vertically, such that the intensity coupled into one of the two fibers can be adjusted with a home-built attenuator assembly. Balanced detection is a very important feature of our spectrometer. We almost always use it for frequency-swept or field-swept experiments. However, balancing is challenging for wavelength-resolved experiments. These problems could be resolved with auto-balancing detectors or by motorizing the attenuator assembly, which would also provide signal-to-noise ratio improvements for long-duration experiments (where detector drift often occurs).

After the fiber, the probe beam is collimated and re-focused onto the sample by a pair of achromatic lenses (a long-pass filter is placed in-between to eliminate residual UV light). The sample mount contains a "field lens" immediately after the sample to improve refocusing characteristics. The diverging beam post-sample is collimated and re-focused, with a pair of achromatic lenses, onto an optical fiber. A 642 nm notch filter (Semrock StopLine) is used to block pump scatter. Additional optical filters are added as needed to prevent spectral contamination from, for example, second order diffraction. The collection fiber is coupled to a photodiode for detection. We have used several detectors. Most experiments used the New Focus 2307 balanced photodetector, because it has a large active area, high sensitivity, switchable gain, and permits balanced detection. Even when we are not balancing, we typically use this detector. These benefits come at the cost of a low bandwidth, so experiments involving high modulation frequencies (e.g. lifetime measurements) are detected with a higher bandwidth detector (Femto balanced photodetector, HBPR-200M-30K-SI-FC). For NIR experiments, we used a New Focus 2317 balanced photodetector.

The photodiode signal, proportional to the transmitted light, $T(t)$, is measured with a lock-in amplifier (Zurich Instruments UHFLI, 600 MHz bandwidth). The AC (modulated) part of this signal ($\propto \Delta T$) is measured by the lock-in amplifier. This lock-in has several built-

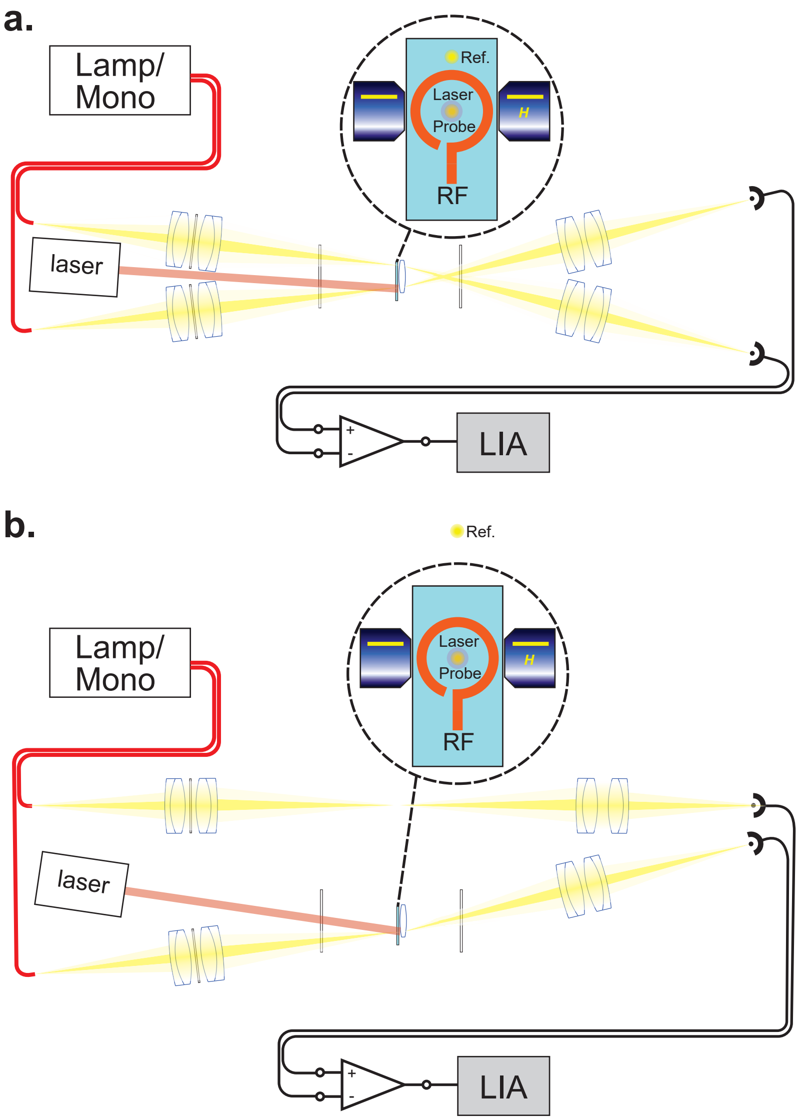

Figure S4: Optical interface at the sample. a. Idealized layout. Narrow-band probe and reference beams are coupled from the same lamp and monochromator into a 1000-micron bifurcated fiber bundle. Relay lens pairs consisting of 75-mm focal length and 80-mm focal length achromats focus the beams onto the sample surface and a reference plane. Inverted identical relays refocus the transmitted beams onto balanced optical receivers for phase sensitive detection. Dielectric long-pass filters in the collimated spaces block the second-order monochromator component. Optical excitation of the sample is from a laser launched via a single-mode fiber. At the sample (inset figure), probe and laser are overlapped within the microwave and static magnetic fields. A 40-mm focal length biconvex field lens at the sample allows improved cryostat access for other optical schemes. **b.** The actual layout does not pass the reference beam through the cryostat or sample, for practical reasons, but is otherwise the same.

in demodulators and two internal reference oscillators, enabling simultaneous measurement of signals at two different modulation frequencies. This can be useful for dual modulation experiments, but also for simultaneous measurements of T and ΔT (T is recorded with high accuracy by setting the reference frequency of the secondary demodulator to zero). But most PADMR experiments do not require a high bandwidth lock-in amplifier with multiple demodulators. For weak signals, we measure T as a separate experiment, because measuring it simultaneously requires either DC coupling at the signal input or splitting the signal. Both of these introduce substantial noise, and the latter can also introduce artifacts. Weak signals are also usually measured at low modulation frequencies. We mostly use the high bandwidth and extra features of the UHFLI for PA experiments and dual-modulation experiments (we rarely use dual-modulation).[4]

The pump laser (Toptica iBeam Smart) is overlapped with the probe beam on the sample, with a spot size of around 1 mm. The 642 nm laser is continuous-wave, but can be digitally modulated at frequencies up to 200 MHz and can provide powers of up to 150 mW.

For PA experiments, the overall bandwidth is limited to about 150-200 MHz; in terms of exponential decay time constants, the IRF corresponds to about a 1.8 ns time constant.

The RF source (Rohde and Schwarz SMB100A, equipped with an extended frequency range option, SMB-112, and the pulse generator option, SMB-K23) can produce a broad frequency range (100 kHz to 12.75 GHz), with maximum output powers of 30 dBm, but above 18 dBm the output is significantly contaminated with harmonics (Fig. S5). Harmonic contamination causes spurious signals at integer fractions of the output carrier frequency. While this is not usually a problem for field-swept experiments at fixed-frequency, it can be. External amplifiers can cause similar problems.

The RF output is connected to the cryostat's RF feedthrough with high-quality hand-formable coaxial cable. The cable quality is crucial for this connection, since the high RF frequencies will leak out of poorly shielded cables, attenuating the RF drive and potentially causing interference via radiated emissions. It is also critical that this cable is as short as

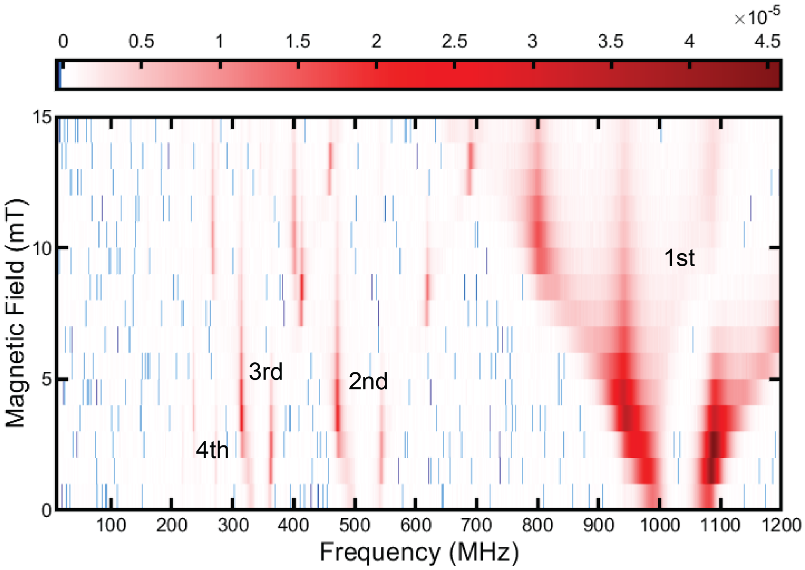

Figure S5: PADMR signal shows harmonic contaminated RF output. This data shows two artifact sources, harmonic contamination and instrument resonances. Instrument resonances are discussed below. The RF source produces high quality output, but when it is run at powers above its specifications, its output is contaminated with harmonics. Signals are seen at the expected locations, but also at 1/2, 1/3, and even 1/4 the expected frequency (because *those* frequencies are contaminated with harmonics).

possible, especially if there are known impedance mismatches. A torque wrench is used for SMA connections.

The RF irradiation is provided by one of two components. First is a home-etched broadband PCB loop antenna, which is pictured in Fig. S2.[5] Second, for field-swept measurements at higher frequency RF drive, is a planar resonator etched on an alumina substrate (~ 10 GHz design frequency). The resonator design balances the maximum RF source output frequency, the maximum attainable magnetic field, optical access and a small footprint, which is limited by the cryostat's radiation shield.

The broadband loop antenna operates well up to at least a few GHz. But as effectively a short-circuit, the impedance mismatch results in resonances at some RF frequencies (Figure S6). This problem is not easily avoidable but is somewhat alleviated by using short transmission lines. We note that these resonances are not always observed, but we are not sure why. Most PADMR data we have collected do not show such obvious resonances as

those in Figure S6. But these data highlight one of the downsides to frequency-swept ODMR measurements — obtaining a completely flat response is not straightforward. Without explicit knowledge of the frequency-response characteristics, care must be taken to not over-interpret signal amplitudes in frequency-swept data. On the other hand, $f \times B$-PADMR is useful for understanding the zero-field to high-field transformation, especially when multiple overlapping signals exist. Although we have not done so, it is possible to take advantage of such resonances. For example, by collecting sensitive field-sweeps at several RF-drive frequencies, the spacing of which should be controllable by varying the coaxial cable length.

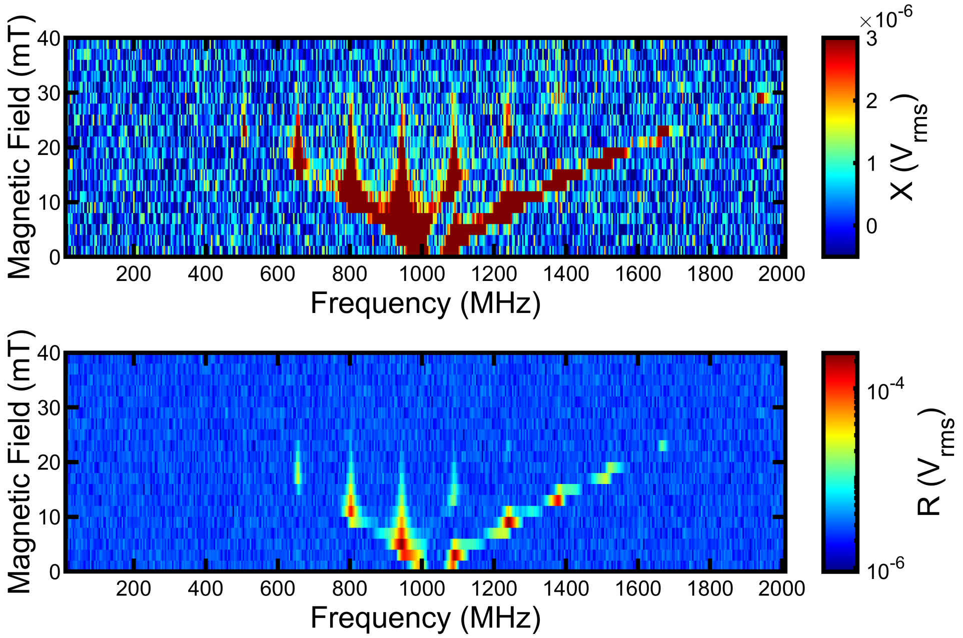

Figure S6: PADMR for a TSPS-PDT film, resolved by magnetic field and RF-frequency sweep ($f \times B$-PADMR) identifies instrument resonances. Upper: The in-phase data, raw. **Lower:** The PADMR signal magnitude, raw, plotted with logarithmic color and z- scales. The PADMR signal presents with sharp peaks spaced by around 145 MHz, corresponding to regions of extremely enhanced sensitivity. These clearly result from instrument resonances, presumably in part from impedance matching issues. The amplification appears approximately Lorentzian, with estimated resonator Q-factors of around 70.

S1.2.4 Experimental Details

All data were recorded at temperature ≈ 5 K, but were likely slightly higher within the probe spot. Wavelength axes were calibrated using the 642 nm notch filter, and the probe source was a tungsten lamp and monochromator. Balancing was not used for PA data, although signals were detected with balanced photodetectors (the reference was blocked). Reported laser powers are as presented by the diode laser's control software, and are about 1.5 times higher than the sample receives. The laser spot diameter has been measured with a beam profiler at 940μm (Gaussian).

PA data

The visible PA spectrum in main text Figure 2b, and the modulation-frequency- and wavelength-resolved PA data in Figures S10, S11 and S12: The ΔT and T data were detected separately with a high bandwidth balanced detector (Femto balanced photodetector, HBPR-200M-30K-SI-FC). The ΔT measurement used 19 mW laser power, 1 kHz laser modulation frequency. For the T measurement, the laser was off and the signal was recorded using the lock-in amplifier, set to 0 Hz reference frequency (the low-pass filter allows much better T measurements than the auxiliary input).

The NIR PA spectrum in main text Figure 2b inset: all details were the same as above except we used a New Focus 2317 germanium balanced photodetector, and an 800 nm long-pass filter was added after the sample.

The laser power dependent PA data in Figure S13: New Focus 2307 balanced photodetector set to low gain (nominal 1 MHz bandwidth, but seems to be closer to 200 kHz). 594 nm probe wavelength (xenon arc lamp source).

The magnetic field dependent PA data in Figures S14 and S15: 594 nm probe wavelength (xenon arc lamp source), 10 mW laser power, using balanced detection and the Femto balanced photodetector.

PADMR data

The f-PADMR data in Figures 3 (main text), S18, S19, and S7: 600 nm probe wavelength, 150 mW CW laser excitation, 2.99 kHz RF modulation frequency (15 dBm source output). Detected with New Focus 2307, balancing was not used. Transmitted light dropped by about a factor of two during the four day experiment, apparently because of increased scattering.

The laser power dependent f-PADMR data in Figure S17: 600 nm probe wavelength, balanced detection, CW laser excitation, 2.99 kHz RF modulation (18 dBm source output), zero-magnetic field.

The $f \times \lambda$-PADMR data in Figures 4 and S20: 150 mW CW laser excitation, 2.99 kHz RF modulation frequency (18 dBm source output). Detected with New Focus 2307, balancing was not used.

S1.2.5 Rephasing Lock-in Signals

In frequency-domain experiments, the phase of a measurement ($\Theta_{obs} = \arctan \frac{Y_{obs}}{X_{obs}}$) takes a central role. For any lock-in signal, an individual measurement will appear with a phase, relative to the lock-in reference. By applying an equal and opposite phase shift to the data, the signal can be redistributed entirely into one channel. This is the post-collection analog of the "auto-phase" button found on lock-in amplifiers. Rephasing the data into a single channel is *not* the same as $R = \sqrt{X^2 + Y^2}$. The easiest way to implement a phase shift starts by first treating each quadrature measurement (X, Y pair) as a single complex number, $S = X + iY$. A phase shift, ϕ, is then applied to the complex-valued signal: $S_{shifted} = S \times e^{i\phi}$. When data are collected at only a single modulation frequency (such as MR or PA spectra), applying such phase shifts can be useful for improving SNR, especially if there are time-delays in the system. The easiest way to estimate the correct phase shift, is to minimize the sum of squares of Y, as a function of the applied phase shift. This could, in principle, be done with least-squares optimization, but we just loop over an array of possible phase shifts. When rephasing data,

if the dynamics are not constant as a function of the scan variable, then there will be no way to redistribute all of the signal into a single channel. Conversely, when there is no value of ϕ that can redistribute all of the signal into a single channel, then scan-variable-dependent dynamics are implicated. Section S1.2.6 demonstrates the data workup and rephasing process for the f-PADMR spectrum of TSPS-PDT. For all data presented in the main text, rephasing did not substantially change the spectral shapes but slightly increased the overall amplitudes and signal-to-noise ratios relative to presenting the in-phase or out-of-phase data alone.

S1.2.6 Data Analysis and Presentation Details

f-PADMR data workup

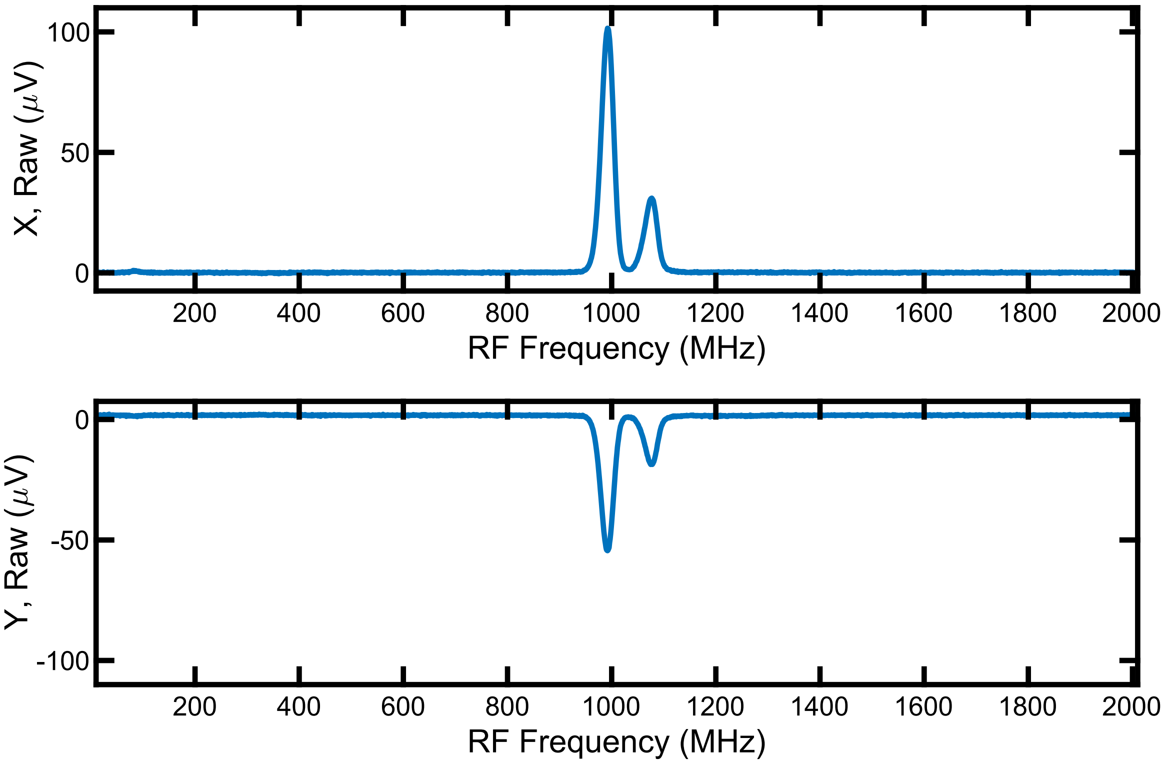

Figure S7: Raw f-PADMR data for TSPS-PDT at zero-field. The raw lock-in amplifier signal for the data in Figure 5 (main text). **Upper:** The in-phase data (X) **Lower:** The out-of-phase data (Y). These raw data show that nearly half of the signal appears in the Y (out-of-phase) channel — it does not strictly obey the steady-state criterion. However, differences between the in-phase and out-of-phase spectra are very small.

The data in Figure 3a (main text) were produced from the raw data in Fig. S7 by the following procedure: (1) Conversion to a complex number — the X and Y data from the 23 scans, collected over 3 days, were averaged and then combined into a single complex-valued array, S. X and Y are the real and imaginary parts, respectively. (2) Background subtraction — the data from 1759 MHz to 2010 MHz are averaged to estimate the background signal, and subtracted from the complex-valued array. (3) Scale the PADMR signal by the value of T — for this data set, the value of T at 600 nm was measured before and after the experiment and averaged. The complex-valued signal was divided by the average value of T to give $\frac{\Delta T}{T}$. Over the course of the experiment, the value of T dropped to about half its initial value due to increased scattering. In choosing to use a single averaged value for T, we assume the overall effect on T associated with RF drive is small. Although it would be preferable to measure ΔT and T simultaneously, doing so substantially reduces the signal-to-noise ratio (SNR). For weak signals (almost any PADMR data, but not usually PA data), it is prohibitive. (4) The data were rephased — the choice to rephase at all is somewhat arbitrary, but slightly improves the SNR, such that one plot may be presented, rather than two. When data are described by a single time constant, or when the steady-state criterion is met, there is a single optimal phase shift. In all other cases, the choice of phase is less clear-cut. For this data set, we found the "optimal" phase by first making an array of phase-values to check, ranging from 0 to 180 degrees with 0.1 degree steps. Looping over all the phase-values, we then applied each phase shift to the complex-valued data set, ($S' = e^{i\phi} \times S$), squared the rephased array, S' element-wise, and summed the resulting array to produce a "figure-of-merit" for each phase value. The phase-shift that produced the smallest sum of squares, 29.8°, was chosen to rephase the data.

The rephased data channels, $X' = Real(S')$ and $Y' = Imag(S')$ are plotted in Figure 3a and 3c (main text). Since the dynamics are not exactly single-exponential, this choice of the phase does not actually succeed in moving all of the signal into one channel, but the "residual" ($= Y'$) is useful, since its existence quickly demonstrates that the dynamics are

RF-frequency dependent.

f×λ-PADMR Data Workup

The near IR f×λ-PADMR data (Main text, Figure 4). (1) Raw $X(f_{Drive}^{RF}, \lambda)$ and $Y(f_{Drive}^{RF}, \lambda)$ data from 26 scans collected over two days were averaged; and converted into a single complex-valued matrix $S(f_{Drive}^{RF}, \lambda) = X_{ave}(f_{Drive}^{RF}, \lambda) + iY_{ave}(f_{Drive}^{RF}, \lambda)$. (2) The complex data matrix $S(f_{Drive}^{RF}, \lambda)$ was then background-subtracted using the average of $S(f_{Drive}^{RF}, \lambda)$ over two off-resonant values of f_{Drive}^{RF} (910 MHz and 915 MHz) as a wavelength-dependent background spectrum $S_{bg}(\lambda)$. This was subtracted from $S(f_{Drive}^{RF}, \lambda)$ for each value of f_{Drive}^{RF}. This assumes that the background signal is wavelength-dependent but does not depend on f_{Drive}^{RF}, which is not necessarily valid but has been experimentally confirmed. (3) The background-corrected complex data $S_{corr}(f_{Drive}^{RF}, \lambda) = S(f_{Drive}^{RF}, \lambda) - S_{bg}(\lambda)$ was then divided by the wavelength-dependent transmittance $T(\lambda)$, (recorded separately using the lock-in set for a 0 Hz reference frequency) to produce the corrected PADMR signal, $S_{PADMR} = -S_{corr}(f_{Drive}^{RF}, \lambda)/T(\lambda)$. Figure 5a shows the magnitude of this, $R = |S_{PADMR}|$, because it is easier to visualize the wide range of signal amplitudes. (4) The normalized PADMR signal, $S_{PADMR} = X_{PADMR} + iY_{PADMR}$, was phase-shifted by $\phi = +34.2°$, to maximally transfer the signal into the real part, X'_{PADMR}. The rephased real part X'_{PADMR} is very similar to X_{PADMR}, but slightly less noisy. (5) X'_{PADMR} was fit with a global fitting algorithm developed in-house and implemented in MATLAB. The algorithm was adapted from code used to treat transient absorption data, in which the nonlinear axis is the time-axis and is typically described by a sum of decaying exponentials. It uses a hybrid approach: nonlinear least-squares is used to fit the nonlinear parameters for a series of four Gaussian terms, and linear regression is used to find the amplitude of each Gaussian at each wavelength. The center frequencies, f_0 for all four were treated as fit parameters, but a single floating line width parameter, σ, was shared. In each iteration, the algorithm computes the nonlinear terms with the current value of the fit parameters, and then uses matrix algebra to compute

an array of best-fit amplitudes: each Gaussian term is associated with an amplitude spectrum that describes that term's wavelength dependence. These "basis spectra" are shown in Figure 4c. A best-fit matrix is constructed by inverting the matrix algebra; the difference between that and the data is the objective function (we use MATLAB's "lsqnonlin" function). Figure 4b shows slices taken from the reconstruction matrix for a few values of f^{RF}_{Drive}. Since it shows reconstructed slices, the main benefit of fitting is to smooth out random noise and more clearly demonstrate the dominant trends; when that is the only goal, a larger number of Gaussian functions and parameters is generally more conservative, since fits using larger basis sets will more closely resemble the original data. When basis spectra are to be interpreted directly, however, it is important to select a physically meaningful set of terms. The choice to use four Gaussian functions was made based on the presence of non-random residuals: the few that are seen are small (Figure S8). Fit parameters: f_0 =946.3, 972.5, 987.1, and 995.2 MHz; shared Gaussian line width, σ = 10.4 MHz. MATLAB's "multistart" is used to increase the likelihood of finding a global optimum solution, in combination with careful and systematic selection of parameter guesses and bounds.

The visible-probe $f \times \lambda$-PADMR data (Figure S20). A similar procedure to the one described above was used to correct and present the data in Figure S20. A constant complex scalar value (the average over both dimensions of the off-resonant data encompassed between 915-930 MHz and between 628-636 nm, 20 points total). These data were not phase-shifted; Panels S20b-d are extracted from a global fit of the real part of the signal, $-\Delta T/T = -X/T$. A good fit to the two-dimensional data set in Figure S20 required a sum of 10 Gaussian functions along the RF frequency axis. The much larger number of terms compared to the NIR data in main text Figure 5 is mainly because both the $D + E$ and $D - E$ transitions are encompassed in this data set. The need for at least six terms is clear based on visual inspection of the integrated MR spectrum (not shown). The abnormal sharpness in the $T_Z \leftrightarrow T_X$ transition is partially responsible as well. To avoid these complexities, we do not present or interpret basis spectra, but opt instead to use the fit as a means of smoothing

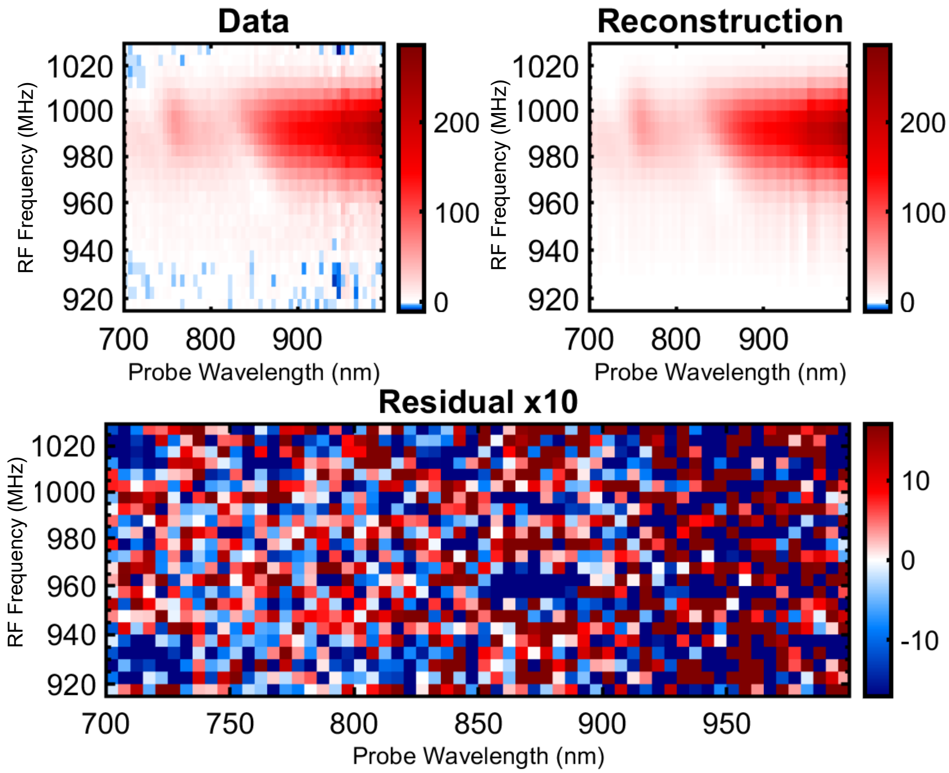

Figure S8: Global fit results for the near-IR $f\times\lambda$-PADMR data. Top left: The in-phase PADMR data after the correction procedure described above (averaging, background subtraction, normalization, and rephasing). **Top right:** The best-fit, or reconstruction, produced by the procedure described above. **Bottom:** The residual, Data - Reconstruction, multiplied by 10 to accentuate non-random residuals (visible areas of red or blue).

data with a physically reasonable model. Hence, we present single traces from the fit, rather than basis spectra.

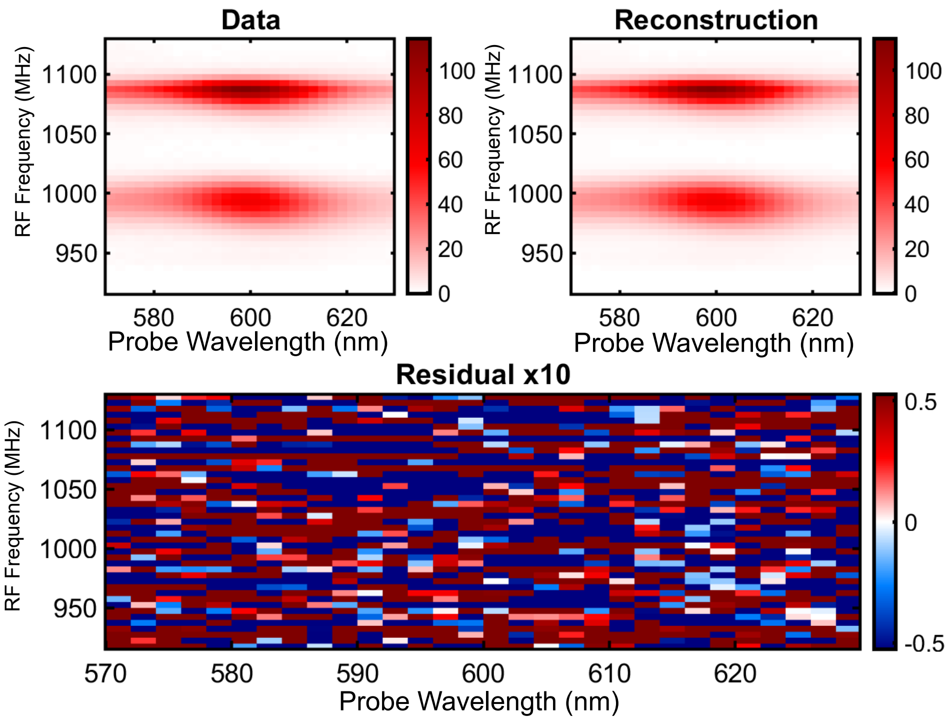

Figure S9: Global fit results for the visible-probe $f \times \lambda$-PADMR data. Top left: The in-phase PADMR data after averaging, background subtraction and normalization (no phase-shift was applied here). **Top right:** The best-fit, or reconstruction, produced by the procedure described above. **Bottom:** The residual, Data - Reconstruction, multiplied by 10 to accentuate non-random residuals (visible areas of red or blue). The minor differences between the fit and raw data are only noticeable in the weakest features, like those in Figure S20d.

S2 Supplementary Discussions

S2.1 PA Spectroscopy of TSPS-PDT

By measuring the PA signal as function of both probe wavelength and modulation frequency, we can measure electronic state dynamics (Fig. S10 and S11). Inspection of both in-phase and out-of-phase PA signals visibly shows that there are at least three distinct components. The most obvious one is associated with the strong PA at 600 nm and relatively long time constants. The second component is associated with a weak PA signal at 475 nm, and is only apparent in the out-of-phase data, where it is not superimposed on the third component. The third component is clearest in the in-phase PA spectra, and shows strong bleach features centered at 478 nm and 535 nm, and PA centered at 550 nm and 610 nm. It does not appear in the out-of-phase data until close to the instrument bandwidth limits.

The PA lifetimes shorten with increased laser pump power (Fig. S13). The lifetime reduction is most likely associated with quenching interactions, but we cannot explicitly rule out increased relaxation rates due to power-dependent sample heating.

The observed lifetimes are also magnetic field-dependent (Figs. S14 and S15). As the field is increased from 0 to 40 mT, the average lifetime is considerably shortened. Since the field range over which this occurs corresponds closely to the experimentally determined ZFS for PDT triplets (see main text and Ref. 1) this must result from sublevel mixing as the zero-field sublevels are transformed under a field. Much less drastic, but still notable, effects are seen in the field range 40 mT to 450 mT. Above 40 mT, the dominant decay rate constant appears to be roughly the average of the three low-field values.

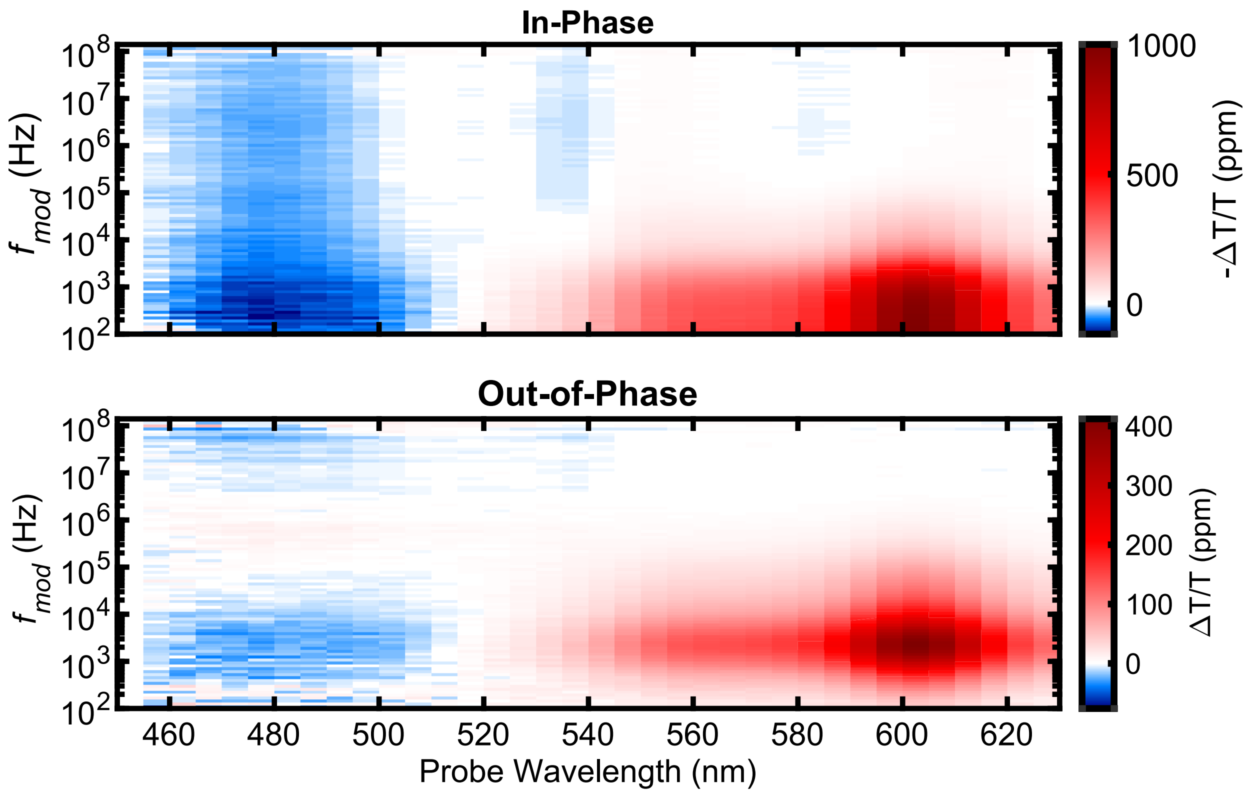

Figure S10: Photoinduced absorption data for a TSPS-PDT film — spectrally resolved electronic-state lifetime measurements. Photoinduced absorption spectra (horizontal slices) as a function of pump modulation frequency (vertical slices). Vertical slices for in-phase signals are described by half of a lorentzian lineshape, (i.e. a lorentzian centered at $f_{mod} = 0$). Vertical slices for out-of-phase signals are described by a dispersion lineshape centered at $f_{mod} = 0$. For single exponential decay dynamics with time constant τ, out-of-phase signals show convenient peaks at $f_{mod} = 1/(2\pi\tau)$ that make lifetime identification very easy. The in-phase and out-of-phase signals are both uniquely useful for data analysis. **Top:** The in-phase channel (X) shows that the strong PA near 600 nm dies off by $f_{mod} = 10$ kHz. Most, but not all, of the bleach between 455 nm-510 nm disappears with the same cutoff frequency. At high modulation frequencies, weak signals are seen that barely register on this scale. **Bottom:** The out-of-phase channel (Y) shows a strong peak at around 2000 kHz. A weak PA is also seen at around 700 kHz/475 nm. Out-of-phase data are negated ($\Delta T/T$) for easier comparison to the in-phase data.

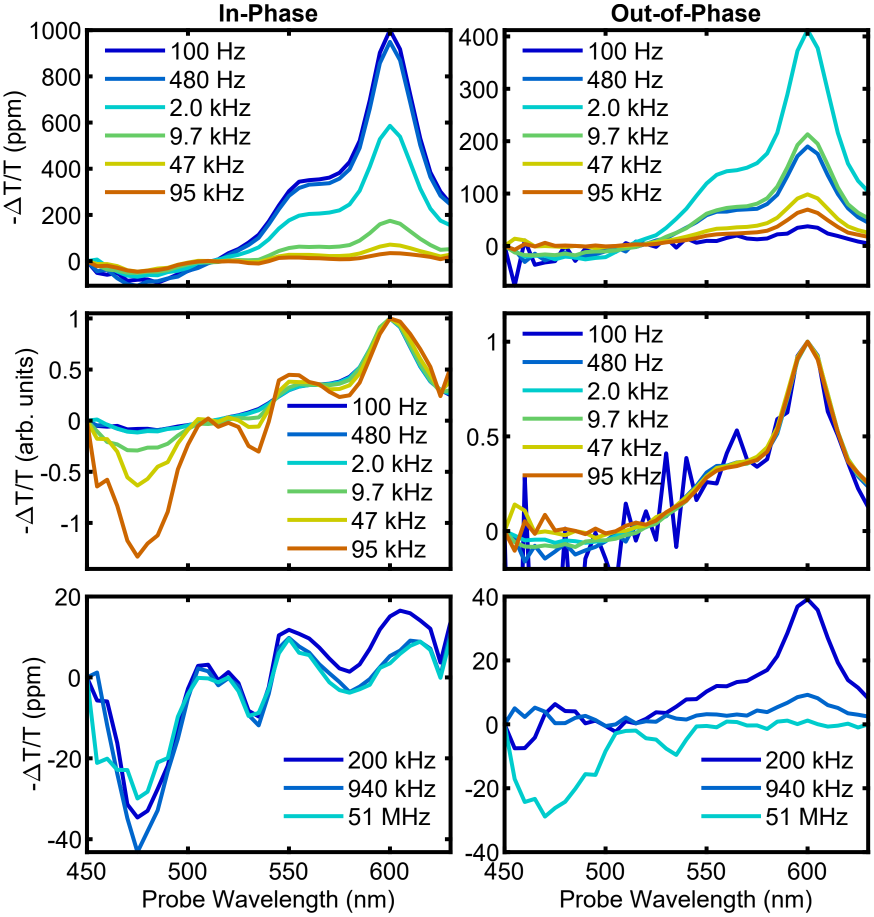

Figure S11: The PA spectra vary with modulation frequency. These are the same data as in Fig. S10. Out-of-phase data are negated ($\Delta T/T$) for easier comparison to the in-phase data. **Top:** PA spectra at low modulation frequencies. **Middle:** A normalized version of the data in the top panels. The out-of-phase PA spectra do not vary much between 100 Hz and 100 kHz modulation frequencies: the bleach centered at 475 nm loses amplitude and becomes a weak PA at modulation frequencies of 47 kHz and 95 kHz (this is more obvious in Fig. S10 between $f_{mod} = 10^5$ and $f_{mod} = 10^6$ Hz). The in-phase PA shows that, at higher modulation frequencies, the strong PA signal at 600 nm no longer contributes to the signal; a very different PA spectrum is seen. This is more obvious in the in-phase data, because in-phase signals are strong at low frequencies, whereas out-of-phase signals are only strong near $f_{mod} = 1/(2\pi\tau)$. **Bottom:** The in-phase PA spectra maintain their amplitude until the instrument bandwidth limits ($\approx$ 100 MHz), suggesting that the response timescale associated with this PA spectrum is instrument response limited ($\tau_{resp} \lesssim 2$ ns). Note that visual comparison of spectral amplitudes associated with different lifetimes is somewhat complicated, because the in-phase and out-of-phase signal amplitudes include factors of τ and τ^2 (Eqs. S1). The small apparent amplitude of the PA spectra at higher modulation frequencies (>50 kHz) is because they have short lifetimes.

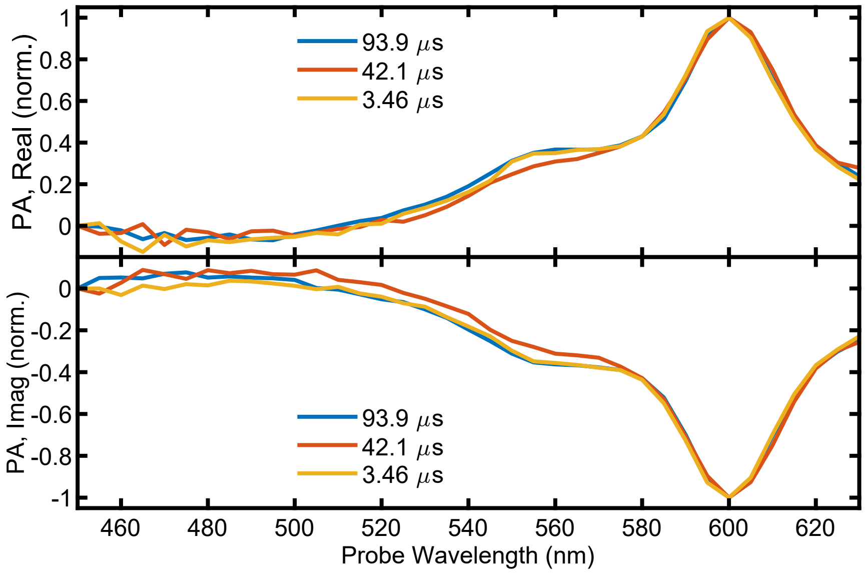

Figure S12: Three lifetimes are associated with nearly identical PA spectra. Spectra are the first three basis spectra from a global fit to the complex data set in Fig. S10. **a.** the real data (in-phase), **b.** the imaginary data (out-of-phase). Minor differences are seen in the relative amplitude of vibronic components, but they are very similar. Out-of-phase data are shown with their raw sign.

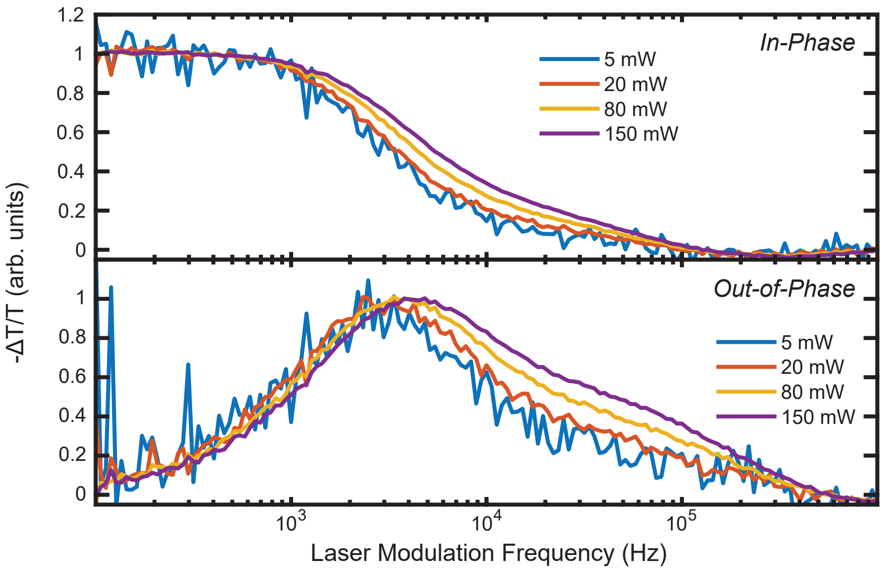

Figure S13: The observed lifetimes are laser-power-dependent. A series of PA lifetime measurements at a range of laser pump powers shows that higher laser powers accelerate the longest decays (600 nm probe). The pump diameter is ≈ 1 mm. Out-of-phase data are shown with opposite sign ($\Delta T/T$) for easier comparison to the in-phase data.

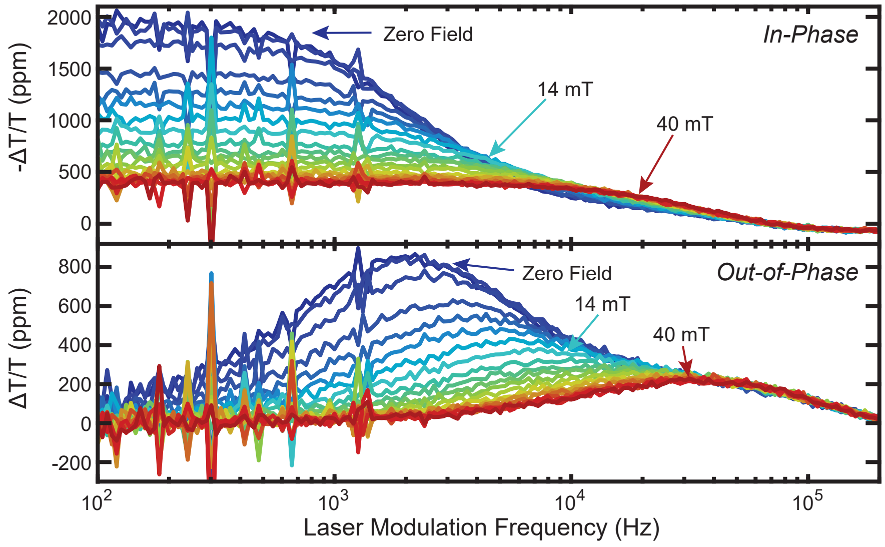

Figure S14: Magnetic fields significantly alter the dynamics. As the static magnetic field is increased, starting from zero, the longest lifetime components are gradually lost. As they are lost, shorter lifetime signals contribute more to the signal. At around 40 mT, which is slightly larger than the triplet ZFS, the dynamics stop varying rapidly with field (Fig. S15). This suggests that sublevel mixing is responsible. Out-of-phase data are shown with opposite sign ($\Delta T/T$) for easier comparison to the in-phase data.

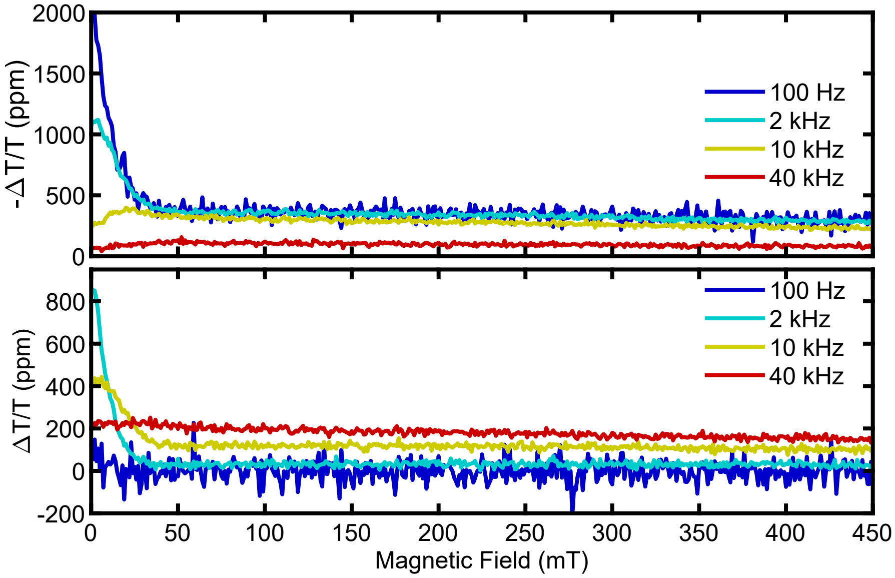

Figure S15: Magnetic field effects at higher fields. Most of the electronic state lifetime perturbations occur at fields below 40 mT. At higher fields, the PA signals at all modulation frequencies vary slowly. Out-of-phase data are shown with opposite sign ($\Delta T/T$) for easier comparison to the in-phase data.

S2.2 f-PADMR Spectroscopy of TSPS-PDT

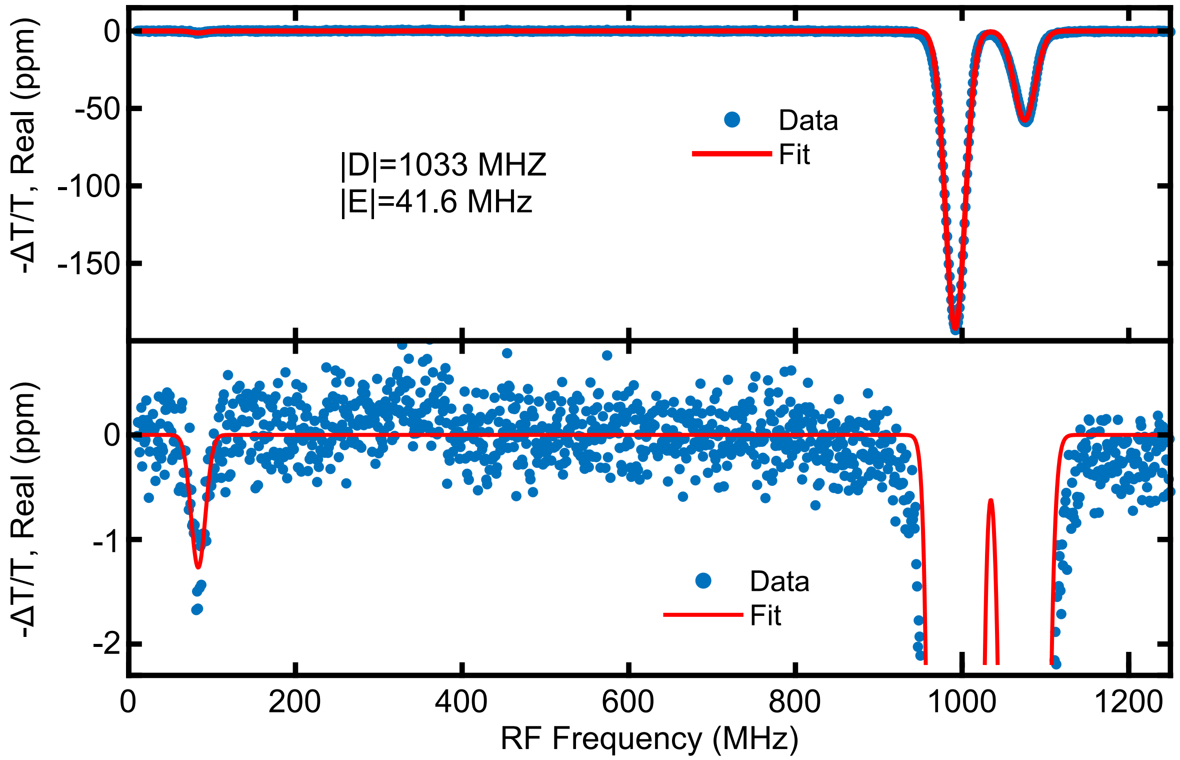

Figure S16: The zero-field f-PADMR spectrum of TSPS-PDT is well-described as a triplet. The fit shown is a sum of three Gaussian functions, $A \exp\left(-\frac{(f-f_0)^2}{2\sigma^2}\right)$, with center frequencies, f_0, of $2E$, $D - E$ and $D + E$. The amplitude, A, and linewidth parameters, σ for each Gaussian were allowed to float independently. Fit parameters: $|D| = 1033$ MHz, $|E| = 41.6$ MHz, $\sigma_{2E} = 8.7$ MHz, $\sigma_{D-E} = 11.8$ MHz, $\sigma_{2E} = 12.6$ MHz. Not described by the fit: weak features at 300-400 MHz and 1150-1250 MHz, and subtle asymmetry in the $D - E$ and $D + E$ line shapes. The weak features are discussed in the main text.

Interestingly, the MR line shape for the 990 MHz peak broadens by about 10 MHz to lower frequencies when the laser intensity is increased (Fig. S17). Although we cannot yet confidently explain this, we imagine that it could be explained by either laser-induced sample heating or increased exciton-exciton interaction frequency.

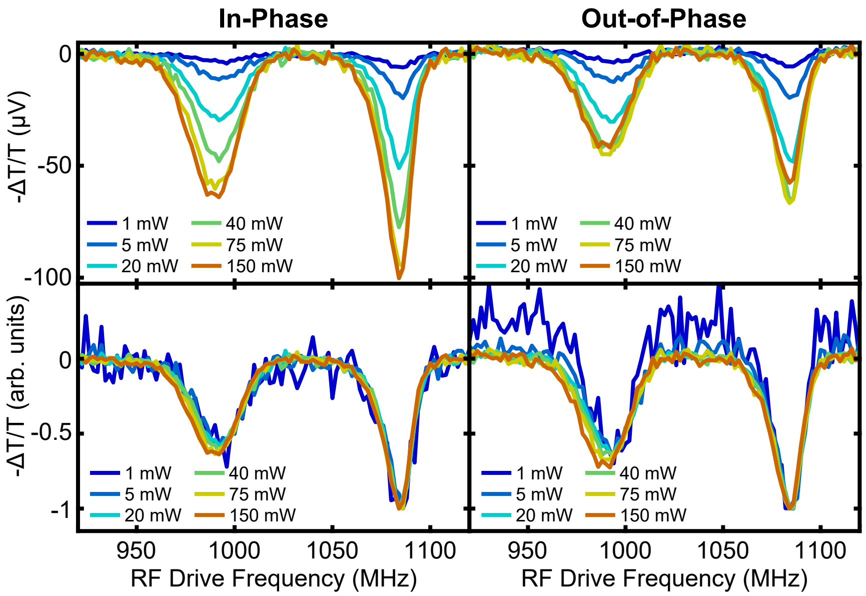

Figure S17: The triplet MR signals broaden when laser power is increased. Top: Raw laser power dependence of the in-phase (left) and out-of-phase (right) PADMR signals. The signals have almost completely saturated by 75 mW laser pump power. **Bottom:** Peak-normalized MR spectra show that the strong MR peak at 990 MHz broadens to its left side. Although the higher frequency MR transition broadens less, if at all, in this experiment that peak was amplified by an instrument resonance (see Section S1.2.3). Since the amplification only occurs within the bandwidth of the instrument resonance, broadening might be suppressed.

S2.2.1 The PADMR signal's frequency-dependent phase

The spectrum in Figure 3a (main text) was measured at a modulation frequency f_{mod} = 2.99 kHz. This is slightly too high to fulfill the steady-state criterion ($f_{mod} \ll \frac{1}{2\pi\tau_{slow}}$, where τ_{slow} is the longest significant signal evolution timescale). Indeed, a significant fraction of the signal appears in the out-of-phase channel. Under true steady-state conditions, the signal phase, θ, does not carry any dynamical information — all signals should appear exclusively in-phase, such that $\theta = 0$. On the other hand, at modulation frequencies comparable to the signal's response bandwidth, the phase gives a small amount of information about the system's dynamics. In the data presented in Figure 3a (main text), the signal phase varies with RF drive frequency (Figures S18 and S19 and Table S1) meaning that different MR transitions are associated with different timescales for the approach to steady state. This is

most evident when comparing the ostensibly $T_Z \leftrightarrow T_Y$ ($\approx$ 990 MHz) and $T_Z \leftrightarrow T_X$ ($\approx$ 1080 MHz) peaks — the latter peak appears with a considerably larger phase, indicating that it is associated with a slower approach to steady-state. Similarly, even *within* those peaks, the phase varies with frequency. For example, the nominally $T_Z \leftrightarrow T_Y$ transition at 990 MHz, cannot be described by a single phase - the low frequency edge is slightly more out-of-phase than the rest of the feature. This suggests a slower sample response on the low frequency edge. Although these phase variations may be related to actual variations in electronic interconversion rate constants, there are other other possible explanations that cannot be ruled out without further experiments. We therefore hesitate to draw specific conclusions about kinetics from these observations.

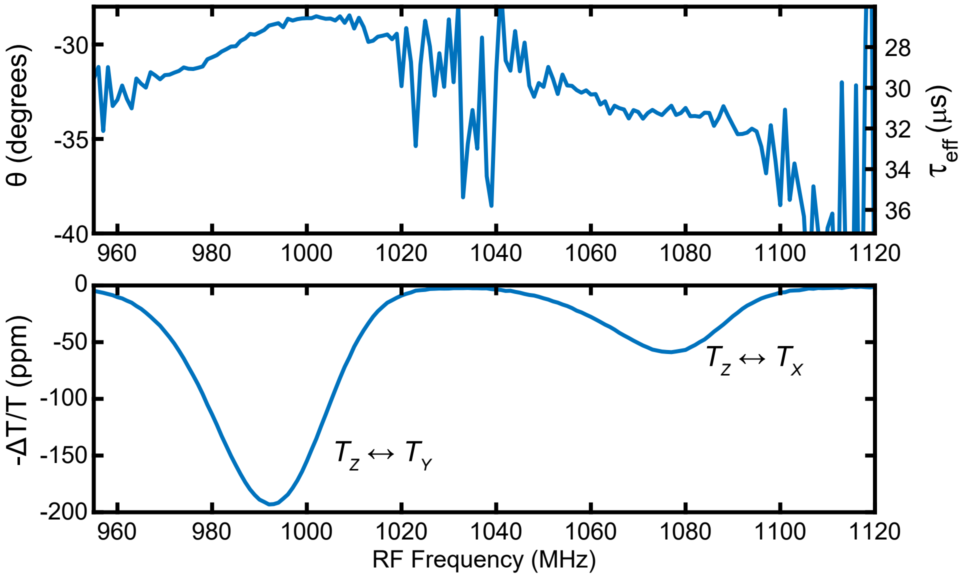

Figure S18: The strong peaks in the f-PADMR spectrum (Figure 3a, main text) are associated with different signal response timescales. In frequency-domain spectroscopy at a single modulation frequency, this manifests as a phase shift that varies with RF drive frequency, which is shown in the top panel. The phase shift (left axis) can be converted to an apparent time constant, $\tau_{app} = \theta/(2\pi f_{mod})$, that roughly corresponds to the steady-state approach timescale (right axis). Note that these values only correspond to actual rate constants in limiting cases. This calculation assumes that the steady-state approach is mainly determined by single exponential dynamics, and that time-delays in the spectrometer are negligible. The latter is probably an acceptable assumption at these relatively low modulation frequencies.

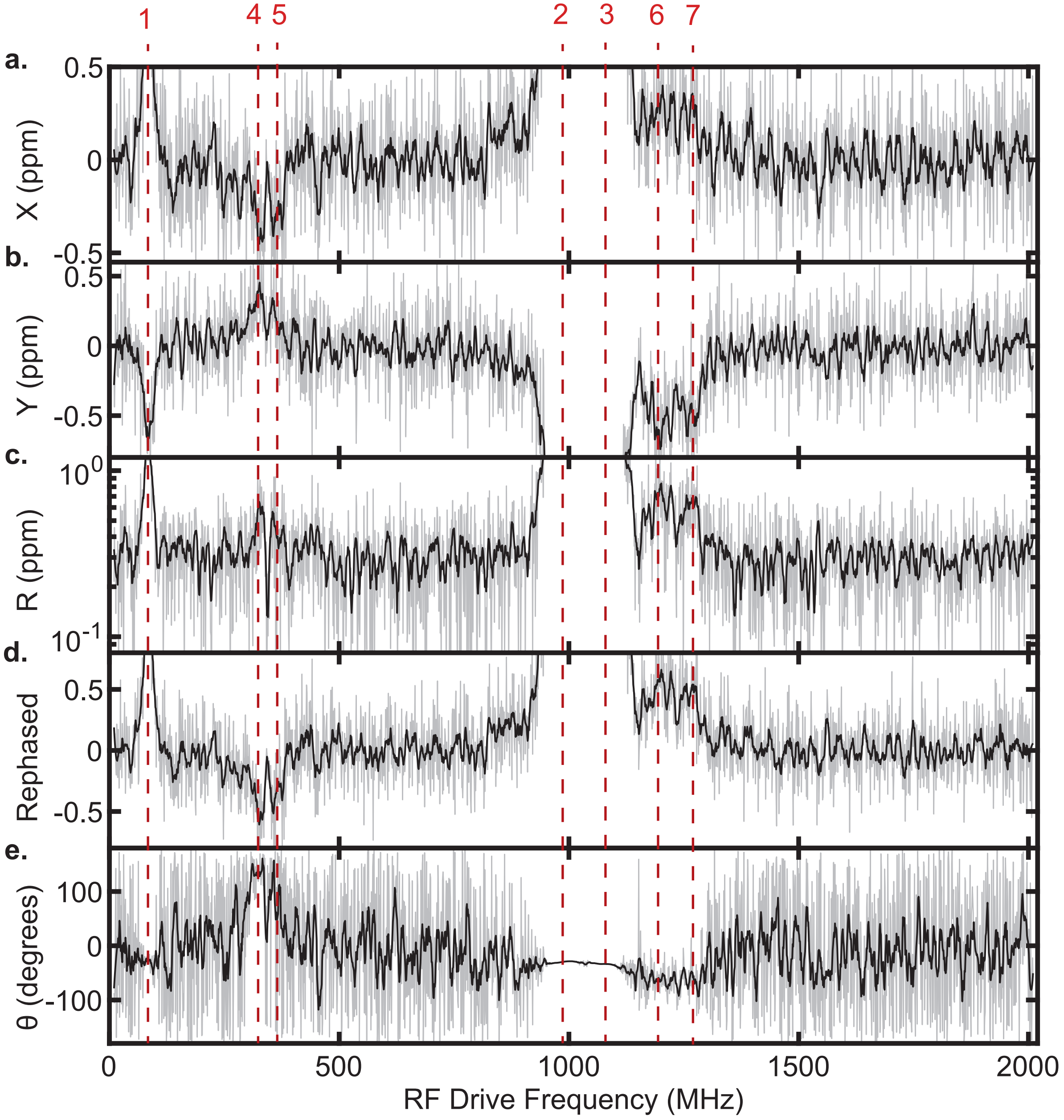

Figure S19: The data set presented are the same as in Fig. S18, but here we focus on the signal near the baseline. Features 1-3 correspond to the triplet transitions. Features 4-7 are assigned in the main text as quintet, but there is some uncertainty about the frequency ranges encompassed by these features and their specific assignments. Gray lines are the raw data after only background correction. Black lines are after smoothing with a 15-point Savitzky-Golay filter. Data was collected every 1 MHz. **a.** X is the in-phase signal, **b.** Y is the out-of-phase signal, **c.** R is the signal magnitude. **d.** The rephased signal is the in-phase data after applying a phase shift of 29.8 degrees. **e.** θ is the phase angle that the signal was detected at. Average phase values for peaks 1-7 are given in Table S1

Table S1: Average phase values and the apparent delay time τ_{app} for peaks 1-7 in Figure S19). Because features 4 and 5 are negative, the measured phase is off by 180 degrees. Numbers in square brackets are the measured phase minus 180 degrees. Peaks 1-3 are triplet transitions. Peaks 4-7 are assigned to ^{5}TT (see text). The value of $\tau_{app} = \frac{\theta}{2\pi f_{mod}}$ does not necessarily correspond to any particular rate constant; it's value roughly relates to the time needed to reach steady-state in the presence of RF drive. That time will be longer when one or both of the states coupled by the RF drive evolve more slowly. It is interesting then, that the ^{5}TT transitions, especially 6 and 7, appear with larger time constants than the T_1 peaks. Uncertainties are 95% confidence intervals computed from the data points averaged for each feature.

Peak Number	Average Phase (Degrees)	τ_{app} (μs)
1	-26±3	24
2	-29.9±0.3	28
3	-33.4±0.2	31
4	139 [-41] ±7	38
5	134 [-46] ±7	43
6	-60 ±5	56
7	-60 ±7	56

S2.3 $f \times \lambda$-PADMR for TSPS-PDT

Assignment of the near IR feature and the probed states. We now discuss the assignments of the λ-PADMR spectra (i.e. the *probed* states). The similarity between the λ-PADMR spectra (Figures 4b and S19) and the PA spectrum (Figure 2b, main text) indicates that the electronic states composing the PA spectrum are also the states most affected by driving the triplet MR transitions. It is natural to expect that driving transitions between the T_1 sublevels should mostly affect the T_1 population. However, the NIR PA features in PDT films have been previously assigned to a triplet-pair state, not T_1.[1] It is possible for the RF-induced population change to manifest more strongly in other electronic states, especially if those states are longer-lived. Further, a change in one electronic state's population must be compensated by an opposite change in at least one other. We therefore individually address each of the four possible assignments for the NIR PA feature: (1) ^{5}TT, (2) ^{3}TT, (3) T_1 and (4) ^{1}TT.

(1) ^{5}TT may be ruled out based on its weak signals in Figure 3a (main text). Since the ^{5}TT sublevels should form with significant spin-polarization and its MR transitions should produce a significant kinetic perturbation,[16] the small signal suggests that its total population is small. This is consistent with our expectation that ^{5}TT is thermally inaccessible at cryogenic temperatures due to strong antiferromagnetic nearest-neighbor exchange.[1] We also expect that ^{5}TT is short-lived (see below), precluding a substantial steady-state population.

(2) A ^{3}TT assignment also seems unlikely. First, we expect very low yields of direct ^{3}TT formation, since its coupling to ^{1}TT is weak.[16–19] While it may also form through re-association of uncoupled triplet states (discussed further below), that is not a high-yield process either. Further, it is thought that ^{3}TT can relax through the triplet-channel annihilation (TCA) pathway $^3\text{TT} \rightarrow \text{T}_1 + \text{S}_0$.[18,20,21] Although the TCA rate is not known, the fact that it is spin-allowed combined with the relatively small energy gap suggest it should be relatively fast.[22] Especially in combination, these factors preclude large steady-state ^{3}TT populations.

(3) Assigning the NIR PA to T_1 would certainly be the simplest explanation, since we have already assigned the MR features in Figure 3a (main text) to it. However, there is little evidence to suggest that T_1 should have such a PA feature. None like it is seen in solution-phase sensitized triplets.[1] While it is conceivable that some optical transitions are only available to T_1 when it is close to another chromophore, such an assignment would also require us to find alternate conclusions for existing reports which convincingly assign similar features in other acene derivatives to ^{1}TT.[23–25]

(4) The final potential explanation is that the NIR PA belongs to ^{1}TT, consistent with those existing assignments.[1,23–25] This assignment appears to be supported by the data. Transient absorption of room temperature TSPS-PDT films, where the NIR PA was first observed, decay with a 47 ns time constant.[1] While that value seems much too short for a T_1 assignment, comparable lifetimes are often observed for spin-allowed relaxation of ^{1}TT in dimers.[26] This argument implies that decay is primarily via $^1\text{TT} \rightarrow \text{S}_0 + \text{S}_0$, since no obvious relaxation on longer timescales that could be attributed to T_1 was seen. This is supported by exchange

computations in Ref. 1: the nearest-neighbor inter-triplet exchange, J_{TT}, was estimated at 2600 GHz, and antiferromagnetic (^{1}TT<^{3}TT<^{5}TT). Although exchange calculations are notoriously difficult, experimental results do confirm that the order of magnitude is plausible.[27] The corresponding thermodynamic barrier to dissociation, $1.5J = 3900$ GHz, is close to the available thermal energy at room temperature (6200 GHz). Although that is a surmountable barrier, it also serves as a weak driving force towards re-association to ^{1}TT. Especially if there is an additional kinetic barrier to triplet hopping, it is reasonable to expect a low probability for ^{1}TT dissociation. At the much lower temperatures used in our experiments, the available thermal energy is much lower than the expected dissociation barrier, strongly arguing that ^{1}TT should be the majority species. Finally, existing models for triplet-pair dynamics are consistent with this explanation (discussed below).

S2.3.1 Probing the 600 nm PA

In Figure S20, we show $f\times\lambda$-PADMR data for TSPS-PDT, meaning that the PADMR signal is measured as a function of RF drive frequency *and* probe wavelength. Vertical slices in the 2D pseudocolor plots correspond to f-PADMR spectra detected at a fixed probe wavelength; horizontal slices are λ-PADMR spectra at a fixed RF drive frequency.

Some skewness is seen in the strong peaks of Figure S20a, meaning that the optical spectrum varies with RF drive frequency. Panels S20b-d are horizontal slices (λ-PADMR spectra) taken from the results of a global fit (see SI). Figures S20c and S20d show the λ-PADMR spectra that result from driving the strong MR peaks. They strongly resemble the qSS PA spectrum of TSPS-PDT (Figure 2b, main text). But increasing RF drive frequencies are associated with blue-shifting PA features. Importantly, the extent of the blue-shift is comparable in both MR features, and occurs over a similar RF frequency range.

The PA blue-shifting as a function of RF drive frequency is not the only interesting feature of the $f\times\lambda$-PADMR data. Figure S20b shows λ-PADMR spectra that result from driving weak MR transitions *near* the strong peaks. 940 MHz RF drive produces a λ-PADMR

spectrum similar to those in Figure S20c,d, although it is noticeably broader than the spectra at any other single RF drive frequency. Weak MR transitions at 1030 MHz and 1130 MHz are both associated with a very different λ-PADMR spectrum (Fig. S20b). At these RF drive frequencies, PA quenching (positive signal) is strongest between 570 nm and 590 nm, and nearly zero at 600 nm.

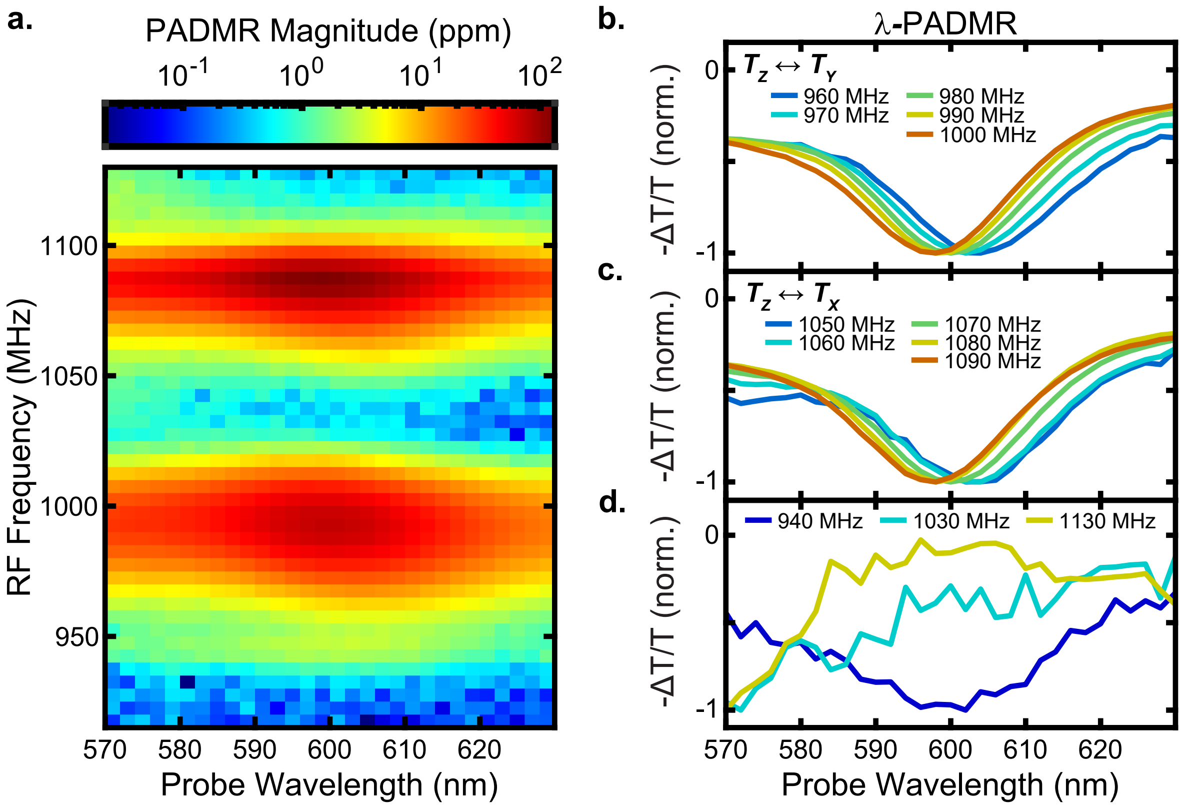

Figure S20: **a.** The PADMR signal magnitude, $R = \sqrt{X^2 + Y^2} = |S|$, demonstrates that the photoinduced absorption signal depends on RF drive frequency. R is used because it permits log scaling of the z-axis and color scale, which enhances the visibility of subtle features. **b-d.** Optical spectra corresponding to specific RF drive frequencies, from a global fit to the real part of the full two-dimensional data set. **b.** The $T_Z \leftrightarrow T_X$ transition shows a blue shift of $\approx$ 7 nm (24 meV = 5800 GHz) as the RF drive frequency is increased from 1050 MHz to 1090 MHz. See text for discussion. **c.** The $T_Z \leftrightarrow T_Y$ transition shows a similar blue shift as the RF drive frequency is increased from 960 MHz to 1000 MHz. **d.** Weak signals visible at 1030 MHz and 1130 MHz show nearly identical PA spectra, in terms of both shape and amplitude, which are clearly distinct from the PA spectra associated with the much stronger transitions at around 980 and 1080 MHz.

S2.3.2 Correlations derive from inhomogeneity

Linear correlations between MR and optical line positions in triplet states (e.g. Fig. S20c,d) have been previously discussed,[28–34] and derive from inhomogeneity of some form. Although the exact nature of the inhomogeneity is not immediately obvious, it must be one that substantially perturbs the electronic environment, or else it would not manifest in both electronic and magnetic resonance line positions.

When two MR transitions can be resolved, it means that the associated state does not rapidly sample the responsible inhomogeneities or else it would lead to line narrowing like is seen with zero-field splittings in solution-phase EPR and NMR spectra. The relevant timescale is defined by the peak-peak frequency separation, $\tau_{Sep} \approx \frac{1}{2\pi f_{Drive}}$. For a 50 MHz frequency separation, we then expect that the inhomogeneities must persist for longer than a few nanoseconds. The most obvious types of inhomogeneity that satisfy this criterion are from sample morphology variations. For example, imperfections like defects, grain boundaries, and amorphous regions. These types of variations will clearly lead to inhomogeneous broadenings if they make up a significant part of the sample and if their excited states do not quickly hop to bulk regions. However, there are other types of inhomogeneity that may be responsible for the observed correlations, if they are persistent. First, multiple pair-wise conformers exist, due to the reduced symmetry of TSPS-PDT relative to true acenes. Second, weak interactions between non-nearest-neighbor triplet-pairs cannot be immediately ruled out. The latter possibility may be supported by broadening in f-PADMR spectra as a function of laser power (Fig. S18), although there are other plausible explanations for that behavior. Unfortunately the possibilities are too difficult to distinguish at this time. Experiments with single crystals or molecular dimers may be able to resolve this uncertainty.

S2.3.3 Optical/MR correlations are normal.

Williamson and Kwiram give the most thorough and comprehensible justification for linear correlations between MR and optical line positions in Ref.[28] In their interpretation, the

inhomogeneity in both MR and optical spectra derive from variations in the local electric potential. The basis set they considered is that of the independent chromophore - the matrix (solvent) is treated separately as a perturbation. Although this assumption clearly does not describe highly π-stacked TSPS-PDT films, it is worth considering their interpretation. Local electric potential variations directly perturb electronic energy levels, whereas they perturb spin-sublevel energies via spin-spin and/or spin-orbit interactions. They argue that SOC is likely insignificant for $\pi\pi*$ triplet states in aromatic hydrocarbons, because they are orbitally non-degenerate (unlike, for example, high-spin octahedral transition metal complexes). In their model, SOC can only occur through higher-lying triplet states of different orbital character. While this assumption is probably acceptable in the systems they were considering, it is not yet clear that they are valid here. The large nuclear charge of sulfur, potentially combined with state mixing from low-lying $n\pi*$, will contribute to spin-orbit coupling — to what extent is not yet clear. Their model suggests that the observed MR line-broadening arises from sublevel-dependent electric dipole moment and polarizability, which themselves derive from spin-orbit and spin-spin coupling effects.

Although we cannot easily distinguish between ZFS caused by SOC vs. SSC, we know based on other aromatic triplet-state molecules that SSC dominates the ZFS. For TSPS-PDT, we have measured $D = 1033$ MHz (see above). We have also measured $D = 1233$ MHZ for the intersystem crossing of TIPS-pentacene in solution,[35] demonstrating that the trend towards reduced D in longer acenes is continued here. We are not aware of ZFS parameters measured for hexacene derivatives, which would be a useful comparison. If sulfur-induced spin-orbit coupling contributed significantly ($\gtrsim 100$ MHz) to the ZFS of TSPS-PDT, we would expect the value of D to deviate from that trend, though it is not clear in which direction. Given that the ZFS measured here is also extremely similar to that found for TIPS-PDT in solution (Fig. S21), it does not appear as if the packing has a large impact on D. This suggests that the local magnetic environment experienced by individual triplet states is very similar (indistinguishable to within ~ 50 MHz) in the mixed iodobutane/toluene solvent system used

there, relative to the film here. Finally, the g_{iso}-factor calculated for that system (2.001) shows a fairly minor deviation from the free electron value, $g_e = 2.0023; \Delta g \approx 650$ ppm. This value carries significant uncertainty due to uncertainty in the g_{iso} measurement (95% confidence interval from the fit Jacobian: $150 - 1100$ ppm). For comparison, Schott et al. reported that the g-shift for TIPS-pentacene radical is 311 ppm, for small thiophene radicals are $\approx 200 - 4000$ ppm (lower values are when symmetry leads to reduced electron density on the sulfur atoms), and for analogous selenophene radicals are $\sim 10,000$ ppm (corresponding to a measured value, $g \approx 1.98$). The relatively small g-shift suggests that internal spin-orbit coupling is not strong. Presumably the fractional electron density is low on the sulfur atoms, since they are a lesser component of a much larger π-system.

Because ground state bleach (GSB) signals only probe excited molecules, bleach wavelengths are a measure of the S_1 energy of the *excited* molecules probed by the PADMR experiment. The lowest energy GSB, corresponds to excited molecules where S_1 is most stabilized. Because it has nominally the same electron configuration, we expect that T_1 is stabilized by the same types of interactions that stabilize S_1, and therefore that it is perturbed in the same direction. This is consistent with the observed correlation in PA and GSB features — when the $S_1 \leftarrow S_0$ transition is low energy, so is the ${}^3T_n \leftarrow {}^3 T_1$ PA. Interestingly, the shift in GSB (~ 90 meV) is roughly three times the shift in the 600 nm PA (~ 24 meV). However, without knowledge of the ${}^3T_n$ energy distortions, we cannot associate the PA shift to a single shifting energy level.

Given that the triplet state may vary in energy by as much as 90 meV, the most heavily stabilized triplet states are likely to act as traps at these low temperatures ($k_BT < 1meV$). But what is the origin of the stabilization? We do not think that amorphous regions, grain boundaries or crystallite surfaces are associated with the low energy transitions, because tight crystal-packing and well-defined crystallites are associated with S_1 stabilization, not destabilization. Crystal-packing strains and impurity-defects cannot be ruled out on the same grounds.

Perhaps the most interesting question is whether or not the inhomogeneous broadening is associated with inter-triplet exchange. Perhaps. First, we can rule out ^{5}TT as the primary source of the MR spectral broadening, because the other, assigned, ^{5}TT transitions are much weaker and seem to evolve on slower timescales (Fig. S1). Therefore, for exchange to be the source of the broadening, then the MR transitions must be from ^{3}TT (with varying triplet-pair interaction strength). For inter-triplet exchange to be the cause of the broadening, it *must* explain the ground state bleach behavior. When a molecule is excited, the corresponding bleach is associated with transitions that cannot occur anymore. The lowest energy bleaches occur for molecules with strong coupling to their neighbors, where the $S_1 \leftarrow S_0$ absorption is most delocalized. If two triplets are situated on strongly coupled, neighboring chromophores, they will experience strong exchange *as well as* showing the lowest energy bleach. It is less obvious whether strongly-coupled triplet-pairs can specifically *cause* the low-energy bleaches due to, for example, prevention of neighboring molecules from engaging in the strongly delocalized optical transitions they are associated with. Clearly, triplet-pairs are not the cause in general of ground state absorption broadening, but that does not preclude triplet-pair interaction strength from being correlated with the optical absorption energy.

Unfortunately the possibilities are too difficult to distinguish at this time. It is possible the MR/optical correlation derives simply from crystal strains or the coexistence of three unique pair-wise conformations. But we cannot yet rule out the possibility that it derives at least partially from ^{3}TT transitions in triplet-pair states with varying degrees of exchange.

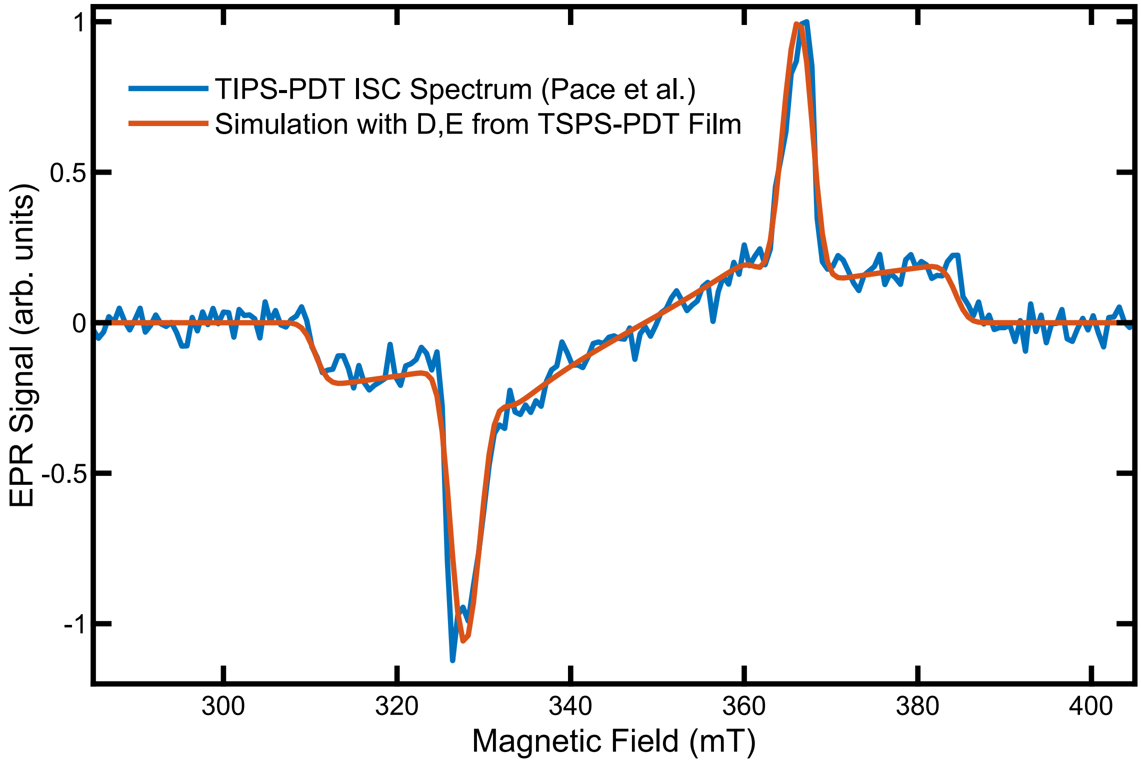

Figure S21: Solution-phase triplet spectrum shows same values of D,E as film, and no evidence of strong spin-orbit coupling. The intersystem crossing spectrum of TIPS-PDT in heavy-atom solvent at 100 K from Ref. 1) (blue line), and a simulation of it (orange line) using the ZFS parameters measured here for TSPS-PDT films ($|D| = 1033$ MHz, $|E| = 41.6$ MHz). Easyspin's pepper function was used for the simulation, with a microwave frequency = 9.740076. Sublevel populations, the g-factor (assumed isotropic), and a Gaussian broadening parameter, Γ, were the only fit parameters used for this simulation. The fit result gave $g_{iso} = 2.001 \pm 0.001$, $\Gamma = 2.3$ mT (6.3 MHz), and relative populations $P_z : P_y : P_x = 0 : 0.05 : 0.95$. The uncertainty for g_{iso} is a 95% confidence interval computed using the fit Jacobian. Note that only *differences* in populations (polarizations) can be interpreted from this data - other experiments or models are needed to determine absolute populations. These populations are notably different than the ones given in Ref. 1, in that they appear to use opposite signs for the value of D — using the values from Ref. 1 gives a similar but inverted spectrum, relative to ours. The value of g_{iso} is similar to most organic triplets — it is close to the free electron value, $g_e = 2.0023$. Deviations of this magnitude ($\Delta g \approx 650$ ppm) are typical of $\pi\pi*$ triplets and confirm that spin-orbit coupling is not particularly strong in solution-phase TSPS-PDT.[36] We note that the field-swept solution spectrum in Ref. 1 does appear to have a slightly larger ZFS than the film.

S2.3.4 RF simulations of the broadband RF coupling loop.

Simulations of the RF coupling loop were conducted in COMSOL Multiphysics v6.0 using the RF module. The coupling loop was fabricated as previously described[5] using a copper coated FR4 board, patterned and etched by hand. The difference in this work is that a central hole is drilled through the board to allow probe light transmission. The simulation represents the pattern as a perfect electrical conductor, and the FR4 as a slab of material with a dielectric constant of $\epsilon_r = 4 - 0.08i$. The end-launch SMP connector is explicitly modeled, and RF energy is introduced via a lumped-port boundary condition at that connection. The mounting hardware and sample substrate were explicitly modeled with the appropriate material properties. The surroundings are represented by perfect electrically conducting walls on the four sides perpendicular to the optical axis, representing the radiation shield in which the sample is housed. The other two ends are partially open electrically due to the optical windows. These are represented in the simulation via "scattering" boundary conditions.

Figure S22 shows the results of these simulations. The coupling loop produces a relatively strong microwave magnetic field in its interior that is perpendicular to the cut plane displayed in Figure S22a. While this field strength depends substantially on frequency, it only declines by 66% from 50 MHz to 1.2 GHz, which is the range of experimental interest in this study. Since the transition rate induced between magnetic sublevels is proportional to the microwave magnetic field, we expect that this also represents a good estimate of how much influence the varying efficiency of the coupling structure could have on the relative intensity of transitions we have observed. It is notable, however, that the field goes nearly to zero at 1.75 GHz, highlighting the need for such simulations to evaluate whether variations in microwave magnetic field strength could substantially distort the observed spectra.

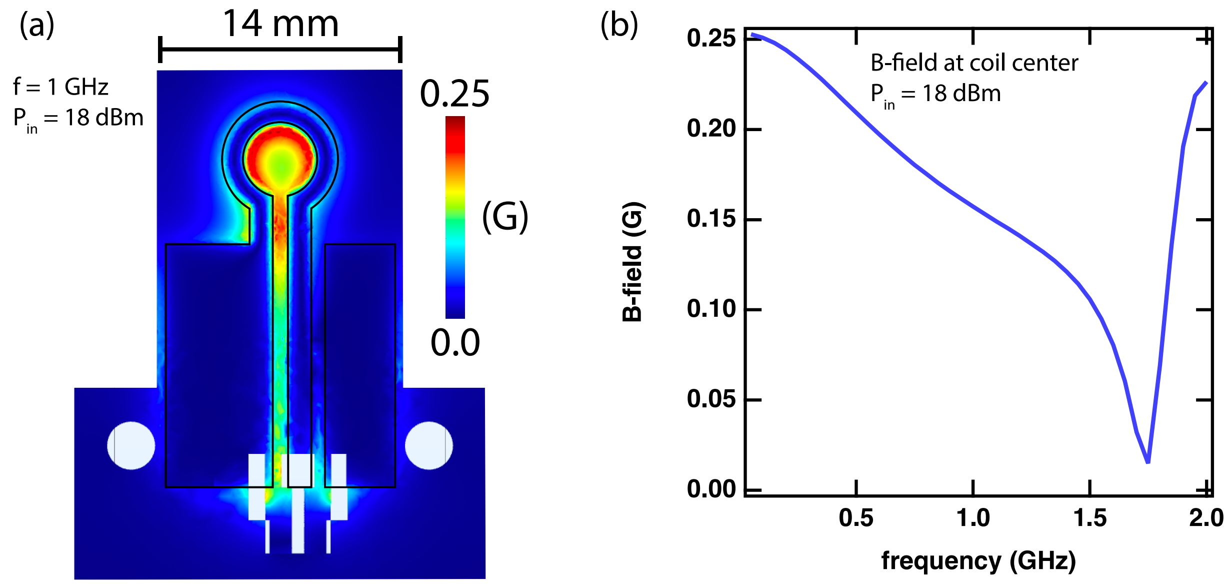

Figure S22: RF simulation results for the low-frequency microwave coupling loop. a. The spatial distribution of absolute microwave magnetic field strength at the sample plane. The field direction is out of the plane. This distribution is for an input power of 18 dBm (63 mW) and a representative frequency of 1 GHz. **b.** Simulation of the microwave magnetic field strength at the center of the loop as a function of microwave/RF frequency.

References

(1) Pace, N. A.; Rugg, B. K.; Chang, C. H.; Reid, O. G.; Thorley, K. J.; Parkin, S.; Anthony, J. E.; Johnson, J. C. Conversion between triplet pair states is controlled by molecular coupling in pentadithiophene thin films. *Chem. Sci.* **2020**, *11*, 7226–7238, doi:10.1039/d0sc02497j.

(2) Wagner, T. W.; Johnson, J. C.; Reid, O. G. Trap-Filling Magnetoconductance as an Initialization and Readout Mechanism of Triplet Exciton Spins. *J. Phys. Chem. Lett.* **2022**, *13*, 9895–9902, doi:10.1021/acs.jpclett.2c02710.

(3) Pace, N. A.; Korovina, N. V.; Clikeman, T. T.; Holliday, S.; Granger, D. B.; Carroll, G. M.; Nanayakkara, S. U.; Anthony, J. E.; McCulloch, I.; Strauss, S. H. et al. Slow charge transfer from pentacene triplet states at the Marcus optimum. *Nat. Chem.* **2020**, *12*, 63–70, doi:10.1038/s41557-019-0367-x.

(4) Dill, R. D. Excited-State Dynamics in Vanadium(II) Polypyridyl Complexes and Singlet Fission Systems: Exploring the Role of Spin with Optical and Magnetic Resonance Spectroscopy. Ph.D. thesis, University of Colorado Boulder, 2022; ProQuest Dissertations Publishing, (Publication ID 2707594033); `https://www.proquest.com/docview/2707594033/`.

(5) Joshi, G.; Dill, R. D.; Thorley, K. J.; Anthony, J. E.; Reid, O. G.; Johnson, J. C. Optical readout of singlet fission biexcitons in a heteroacene with photoluminescence detected magnetic resonance. *J. Chem. Phys.* **2022**, *157*, 164702, doi:10.1063/5.0103662.

(6) Kallel, H.; Latini, G.; Paquin, F.; Rinfret, R.; Stingelin, N.; Silva, C. Background-free quasi-steady-state photoinduced absorption spectroscopy by dual optical modulation. **2010**, doi:10.48550/arXiv.1007.3035.

(7) Howard, I. A.; Mauer, R.; Meister, M.; Laquai, F. Effect of morphology on ultrafast free carrier generation in polythiophene:fullerene organic solar cells. *J. Am. Chem. Soc.* **2010**, *132*, 14866–14876, doi:10.1021/ja105260d.

(8) Lakowicz, J. R. In *Princ. Fluoresc. Spectrosc.*, 3rd ed.; Lakowicz, J. R., Ed.; Springer US: Boston, MA, 2006.

(9) Clarke, R. H., Ed. *Triplet State ODMR Spectroscopy: Techniques and Applications to Biophysical Systems*; John Wiley and Sons, Inc., 1982.

(10) Hoff, A. J. In *Advanced EPR: Applications in Biology and Biochemistry*, 1st ed.; Hoff, A. J., Ed.; Elsevier Science Publishers B.V.: Amsterdam, 1989.

(11) Charles P. Poole, J. *Electron Spin Resonance: A Comprehensive Treatise on Experimental Techniques*, 2nd ed.; John Wiley and Sons: New York, 1983.

(12) Yang, C. G.; Ehrenfreund, E.; Wohlgenannt, M.; Vardeny, Z. V. Comment on "Frequency response and origin of the spin-$\frac{1}{2}$ photoluminescence-detected magnetic resonance in a π-conjugated polymer". *Phys. Rev. B* **2007**, *75*, 246201, doi:10.1103/PhysRevB.75.246201.

(13) Lee, S.-Y.; Paik, S.; McCamey, D. R.; Boehme, C. Modulation frequency dependence of continuous-wave optically/electrically detected magnetic resonance. *Phys. Rev. B* **2012**, *86*, 115204, doi:10.1103/PhysRevB.86.115204.

(14) Stachowitz, R.; Schubert, M.; Fuhs, W. Frequency-resolved spectroscopy and its application to low-temperature geminate recombination in a-Si:H. *Philos. Mag. B* **1994**, *70*, 1219–1230, doi:10.1080/01418639408240285.

(15) Goovaerts, E. Optically detected magnetic resonance (ODMR). *eMagRes* **2017**, *6*, 343–358, doi:10.1002/9780470034590.emrstm1524.

(16) Smyser, K. E.; Eaves, J. D. Singlet fission for quantum information and quantum computing: the parallel JDE model. *Sci. Rep.* **2020**, *10*, 18480, doi:10.1038/s41598-020-75459-x.

(17) Benk, H.; Sixl, H. Theory of two coupled triplet states. *Mol. Phys.* **1981**, *42*, 779–801, doi:10.1080/00268978100100631.

(18) Merrifield, R. E. Magnetic effects on triplet exciton interactions. *Pure Appl. Chem.* **1971**, *27*, 481–498, doi:10.1351/pac197127030481.

(19) Johnson, R. C.; Merrifield, R. E. Effects of Magnetic Fields on the Mutual Annihilation of Triplet Excitons in Anthracene Crystals. *Phys. Rev. B* **1970**, *1*, 896–902, doi:10.1103/PhysRevB.1.896.

(20) Chen, M., et al. Quintet-triplet mixing determines the fate of the multiexciton state produced by singlet fission in a terrylenediimide dimer at room temperature. *Proc. Natl. Acad. Sci.* **2019**, *116*, 8178–8183, doi:10.1073/pnas.1820932116.

(21) Groff, R.; Merrifield, R.; Avakian, P. Singlet and triplet channels for triplet-exciton fusion in anthracene crystals. *Chem. Phys. Lett.* **1970**, *5*, 168–170, doi:10.1016/0009-2614(70)80033-1.

(22) Bixon, M.; Jortner, J. Intramolecular Radiationless Transitions. *J. Chem. Phys.* **1968**, *48*, 715–726, doi:10.1063/1.1668703.

(23) Tuan Trinh, M.; Pinkard, A.; Pun, A. B.; Sanders, S. N.; Kumarasamy, E.; Sfeir, M. Y.; Campos, L. M.; Roy, X.; Zhu, X. Y. Distinct properties of the triplet pair state from singlet fission. *Sci. Adv.* **2017**, *3*, 1–9, doi:10.1126/sciadv.1700241.

(24) Miyata, K.; Conrad-Burton, F. S.; Geyer, F. L.; Zhu, X. Y. Triplet Pair States in Singlet Fission. *Chem. Rev.* **2019**, *119*, 4261–4292, doi:10.1021/acs.chemrev.8b00572.

(25) Khan, S.; Mazumdar, S. Theory of Transient Excited State Absorptions in Pentacene and Derivatives: Triplet-Triplet Biexciton versus Free Triplets. *J. Phys. Chem. Lett.* **2017**, *8*, 5943–5948, doi:10.1021/acs.jpclett.7b02748.

(26) Gilligan, A. T.; Miller, E. G.; Sammakia, T.; Damrauer, N. H. Using Structurally Well-Defined Norbornyl-Bridged Acene Dimers to Map a Mechanistic Landscape for Correlated Triplet Formation in Singlet Fission. *J. Am. Chem. Soc.* **2019**, *141*, 5961–5971, doi:10.1021/jacs.9b00904.

(27) Bayliss, S. L.; Weiss, L. R.; Mitioglu, A.; Galkowski, K.; Yang, Z.; Yunusova, K.; Surrente, A.; Thorley, K. J.; Behrends, J.; Bittl, R. et al. Site-selective measurement of coupled spin pairs in an organic semiconductor. *Proc. Natl. Acad. Sci. U. S. A.* **2018**, *115*, 5077–5082, doi:10.1073/pnas.1718868115.

(28) Williamson, R. L.; Kwiram, A. L. On the nature of the relationship between inhomogeneously broadened phosphorescence and ODMR spectral lines. *J. Chem. Phys.* **1988**, *88*, 6092–6106, doi:10.1063/1.454502.

(29) Loboda, O.; Minaev, B.; Vahtras, O.; Rudd, K.; Agren, H. Ab initio study of nonhomogeneous broadening of the zero-field splitting of triplet guest molecules in diluted glasses. *J. Chem. Phys.* **2003**, *119*, 3120–3129, doi:10.1063/1.1589732.

(30) Gradl, G.; Friedrich, J. Investigation of inhomogeneous line broadening of ODMR transitions in doped organic glasses. *Chem. Phys. Lett.* **1985**, *114*, 543–546, doi:10.1016/0009-2614(85)85138-1.

(31) Clark, S.; Tinti, D. Relationship of the inhomogeneous broadenings in the triplet state optical and ODMR spectra of NaNO2/Ag+. *Chem. Phys.* **1980**, *51*, 17–30, doi:10.1016/0301-0104(80)80076-0.

(32) Kwiram, A. L.; Alexander Ross, J.; Deranleau, D. A. Wavelength dependence of the zero-field splittings in the triplet state of tryptophan. *Chem. Phys. Lett.* **1978**, *54*, 506–509, doi:10.1016/0009-2614(78)85272-5.

(33) Lemaistre, J.; Zewail, A. On the inhomogeneous and homogeneous broadenings of optical and ODMR transitions of triplet states in solids. *Chem. Phys. Lett.* **1979**, *68*, 296–301, doi:10.1016/0009-2614(79)87204-8.

(34) Van Egmond, J.; Kohler, B. E.; Chan, I. Solvent shift broadening in the PMDR spectrum of quinoxaline. *Chem. Phys. Lett.* **1975**, *34*, 423–426, doi:10.1016/0009-2614(75)85529-1.

(35) Dill, R. D.; Smyser, K. E.; Damrauer, N. H.; Eaves, J. D. Entangled , Spin-polarized Excitons from Singlet Fission in a Rigid Dimer. **2022**,

(36) Schott, S.; McNellis, E. R.; Nielsen, C. B.; Chen, H. Y.; Watanabe, S.; Tanaka, H.; McCulloch, I.; Takimiya, K.; Sinova, J.; Sirringhaus, H. Tuning the effective spin-orbit coupling in molecular semiconductors. *Nat. Commun.* **2017**, *8*, 1–10, doi:10.1038/ncomms15200.
